# Understanding the Control of Singlet-Triplet Splitting for Organic Exciton Manipulating: A Combined Theoretical and Experimental Approach

**DOI:** 10.1038/srep10923

**Published:** 2015-07-10

**Authors:** Ting Chen, Lei Zheng, Jie Yuan, Zhongfu An, Runfeng Chen, Ye Tao, Huanhuan Li, Xiaoji Xie, Wei Huang

**Affiliations:** 1 Key Laboratory for Organic Electronics and Information Displays & Institute of Advanced Materials, Jiangsu National Synergistic Innovation Center for Advanced Materials, Nanjing University of Posts & Telecommunications, 9 Wenyuan Road, Nanjing 210023, China; 2 Key Laboratory of Flexible Electronics & Institute of Advanced Materials, Jiangsu National Synergistic Innovation Center for Advanced Materials, Nanjing Tech University, 30 South Puzhu Road, Nanjing 211816, China

## Abstract

Developing organic optoelectronic materials with desired photophysical properties has always been at the forefront of organic electronics. The variation of singlet-triplet splitting (Δ*E*_ST_) can provide useful means in modulating organic excitons for diversified photophysical phenomena, but controlling Δ*E*_ST_ in a desired manner within a large tuning scope remains a daunting challenge. Here, we demonstrate a convenient and quantitative approach to relate Δ*E*_ST_ to the frontier orbital overlap and separation distance *via* a set of newly developed parameters using natural transition orbital analysis to consider whole pictures of electron transitions for both the lowest singlet (S_1_) and triplet (T_1_) excited states. These critical parameters revealed that both separated S_1_ and T_1_ states leads to ultralow Δ*E*_ST_; separated S_1_ and overlapped T_1_ states results in small Δ*E*_ST_; and both overlapped S_1_ and T_1_ states induces large Δ*E*_ST_. Importantly, we realized a widely-tuned Δ*E*_ST_ in a range from ultralow (0.0003 eV) to extra-large (1.47 eV) *via* a subtle symmetric control of triazine molecules, based on time-dependent density functional theory calculations combined with experimental explorations. These findings provide keen insights into Δ*E*_ST_ control for feasible excited state tuning, offering valuable guidelines for the construction of molecules with desired optoelectronic properties.

The ultimate challenge in manipulating conjugated molecules^1^,^2^ for optoelectronic applications is to develop universal approaches capable of controlling excited states for efficient electron-light conversions, affording not only conventional fluorescence^3^ and phosphorescence^4^, but also many other photophysical phenomena including triplet-triplet annihilation (TTA)^5^, singlet fission (SF)^6^, and thermally activated delayed fluorescence (TADF)^7^. The rich photophysical properties of organic molecules have led to many revolutionary developments in organic electronics^8^. Notably, the TTA compounds, which can harvest one singlet exciton from two low-lying triplet excitons, can benefit OLEDs with improved external quantum efficiency (EQE) theoretically up to 12.5% by harvesting the 75% electronically generated triplet excitons to produce singlet excitons for fluorescence^9^; the SF process, which transforms a singlet exciton into two triplet excitons on neighboring molecules with EQE up to 200%, is especially attractive for solar cells in providing doubled photocurrent from high-energy photons^10^; the recently developed TADF materials by harvesting 100% triplet excitons *via* reversed intersystem crossing have achieved EQEs of 20.6% in blue and 30.0% in green TADF OLED devices^11^,^12^, which are comparable to the heavy metal-based phosphorescent emitters^13^,^14^.

To control the triplet/singlet excited states in a designed manner for a desired optoelectronic property, the rational adjustment of the singlet-triplet energy gap (Δ*E*_ST_) between the first singlet (S_1_) and triplet (T_1_) excited states is the key. Typically in Fig. 1, when Δ*E*_ST_ normally laid between 0.5 and 1.0 eV^7^ in conventional compounds is reduced (Δ*E*_ST_ ≤ 0.37 eV)^15^, TADF could be resulted *via* activated endothermic RISC process from T_1_ to S_1_ by the thermal motions of the molecule atoms for the *E*-type delayed fluorescence^16^. Meanwhile, when the Δ*E*_ST_ is increased and the energy of two triplet excitons are close to, or larger than, one singlet exciton (*E*_T1_/*E*_S1_ ≳ 0.5), TTA could happen between triplet exciton interaction pair following the spin statistics rule^17^. SF process, either isoergic or slightly exoergic in producing two triplet excitons with a net spin of zero, is spin-allowed and favorable for fast generation of doubled triplet excitons from high-lying singlet excitons, when S_1_ excitation energy is comparable with twice the energy of T_1_ excitation (*E*_T1_/*E*_S1_ < 0.5)^18^.

Extensive efforts have been so far devoted to reducing Δ*E*_ST_ via separated the highest occupied molecular orbital (HOMO) and the lowest unoccupied molecular orbital (LUMO) strategy to construct efficient TADF molecules^8^. In contrast, TTA and SF molecules were designed by enhancing HOMO-LUMO overlap to the maximum possible degree with enlarged Δ*E*_ST_^18^,^19^. Consequently, efficient TADF compounds were generally found in donor-acceptor (D-A) molecules^20^, while TTA and SF compounds were mostly observed in alternant hydrocarbons with an even number of carbons in conjugated close-shell S_0_ systems^18^. Considering the four-electron picture transformation of SF process, the involvement of charge transfer (CT) character through either inter- or intra-molecular D-A interactions is also crucial for the ultrafast fission^21^,^22^. However, despite these advances to date, it remains a challenge to rationally manipulate Δ*E*_ST_ in a large scale *via* subtle molecular structure adjustments to produce energy levels applicable not only for TADF but also for TTA and SF processes.

The lack of quantitative means in descripting HOMO-LUMO overlap and separation extents should be a main obstacle in establishing accurate relations between Δ*E*_ST_ and molecular structures. Here, we demonstrate a convenient approach in quantifying the frontier orbital overlap and separation with a set of new parameters in both S_1_ and T_1_ states. With the aid of these quantitative parameters, we proposed a molecular symmetry controlling strategy to fine tune the excited state energy levels for accommodation of excitons with diversified spin states and for the support of their varied excited state transfer processes following corresponding photophysical mechanisms of TADF, TTA, and SF. In a typical example demonstrated in 1,3,5-triazine-based molecules, we designed a series of symmetric and asymmetric triazines and successfully realized a widely varied Δ*E*_ST_ in a range from ultralow (0.0003 eV) for TADF and extra-large (1.47 eV) for SF according to time-dependent density functional theory (TD-DFT) calculations and experimental measurements of selectively synthesized molecules. The widely-tuned Δ*E*_ST_ *via* a subtle symmetric control in a uniform molecular architecture is attractive not only for providing a practical guide for material design of TADF, TTA and SF processes, but also for developing a better understanding of the factors that influence the energy levels and spin states of the excited states of organic optoelectronic molecules.

## Results

### Theoretical considerations

The lowest singlet-triplet splitting (Δ*E*_ST_) between the molecular energies at the lowest singlet (*E*_S1_) and triplet (*E*_T1_) excited states were equal to twice of the electron exchange energy (*J*) as illustrated in equation (1) and (2), where *J* is determined by the spatial separation and overlap extents of HOMO (*φ*_H_) and LUMO (*φ*_L_)^23^. From equation (3), the Δ*E*_ST_ is closely related to the frontier orbital overlap extent and separation distance at S_0_ state; higher overlap of HOMO and LUMO and smaller spatial separation (*r*_1_ – *r*_2_) lead to higher *J* and Δ*E*_ST_.


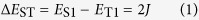



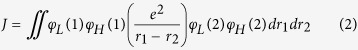



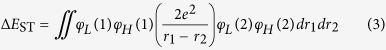



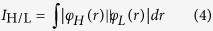



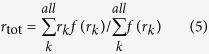



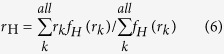



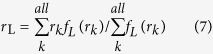



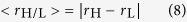


The orbital overlap extent (*I*_H/L_) between HOMO (H) and LUMO (L) can be calculated using the overlap integral function of Multiwfn (eq. (4))^24^. Mean separation distance (<*r*_H/L_>) of HOMO and LUMO can be obtained from the barycenter (*r*_tot_) of the absolute value of the corresponding molecular orbitals (equation (5, 6, 7, 8))^25^. Similarly, the overlap extent and mean separation distance between the highest occupied natural transition orbitals (HONTOs) and the lowest unoccupied natural transition orbitals (LUNTOs) at both S_1_ (*I*_S_ and <*r*_S_>) and T_1_ (*I*_T_ and <*r*_T_>) states were also calculated to give a full-picture analysis of the factors that influence Δ*E*_ST_. The detailed definitions and calculations of Δ*E*_ST_, *I*_H/L_, *I*_S_, *I*_T_, <*r*_H/L_>, <*r*_S_> and <*r*_T_> were presented in Supplementary Information.

### Molecular design

**Figure 2:**
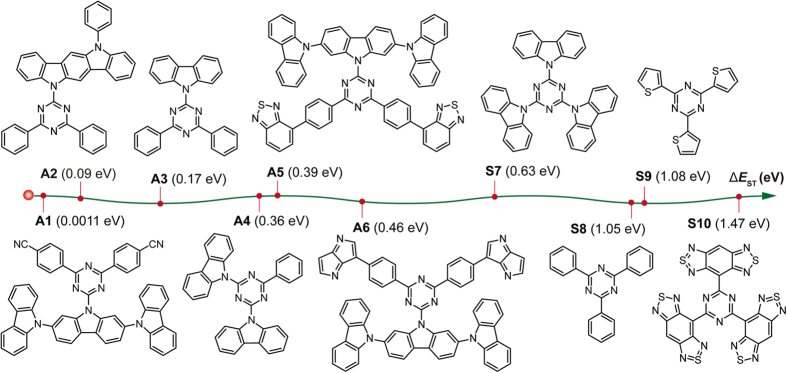
Molecular structures and Δ*E*_ST_ of asymmetric (A1–A6) and symmetric (S7–S10) triazines.

Triazine, which possesses high electron affinity and good thermal stability, is chosen as a basic building block to demonstrate our Δ*E*_ST_ tuning strategy for achieving varied photophysical behaviors of TADF, TTA, and SF in a uniform molecular architecture with symmetry control. The symmetric or asymmetric substitution of various donors or acceptors on three reactive sites of 1,3,5-triazine have resulted in a large number of triazine-based molecules with varied optoelectronic properties^26^. The competition and coordination effects between the substituents, triazine core, and the D-A molecular architecture significantly tuned the molecular energy structures for the singlet and triplet excitons transitions when excited either optically or electronically, and thus leading to rich and/or exceptional optoelectronic properties^27^. Here, we designed a series of triazine-based molecules bearing various donors and acceptors substituted asymmetrically (labeled as **A1**, etc.) and symmetrically (labeled as **S7**, etc.) at three substitution sites of the triazine core (Fig. 2, S1 and S2). Triazines of **A2**, **A3**, and **A4**, were experimentally investigated in the literature^27^,^28^, while **S9** and **A15** were synthesized in this study (Scheme S1) and their properties were measured to verify the computational results.

### Singlet-triplet splitting (Δ*E*_ST_)

As a key parameter in determining the exciton migration and population on excited states, Δ*E*_ST_ is of the most importance^8^. To choose an optimal calculation approach to evaluate Δ*E*_ST_, TD-DFT methods including B3LYP, PBE0, BMK, M062X, M06HF, and long-range correction functionals (ωB97XD and CAM-B3LYP) at 6–31G(d) basis set level were tested. Compared to the experimental Δ*E*_ST_ values (Fig. S3 and Table 1), it is clear that B3LYP gives the best prediction of Δ*E*_ST_ not only for molecules with small Δ*E*_ST_ but also those with large Δ*E*_ST_. In the following investigations, B3LYP/6-31G(d) was selected to predict Δ*E*_ST_ of the designed compounds that lack experimental explorations. The calculated Δ*E*_ST_ demonstrate that the asymmetric triazines have small Δ*E*_ST_ ranging from 0.001 to 0.46 eV, while the symmetric triazines show high Δ*E*_ST_ up to 1.47 eV. Thus, a wide controlling range of Δ*E*_ST_ from almost zero to 1.47 eV has been successfully realized in a uniform molecular system by adopting the conventional symmetry control of triazine substituents (Scheme S2).

The origin of the different effects of symmetric and asymmetric substituents on triazines was investigated *via* frontier orbital analysis. Theoretically, the HOMO is dominated by donor moiety while the LUMO is by acceptor moiety^29^. As a result, the strong donors of tricarbazole and indolocarbazole substituents generally lead to high-lying HOMOs; the strong acceptors of benzonitrile, benzothiazole, and pyrrolo[3,2-b]pyrrole lead to low-lying LUMOs^30^; the LUMOs and HOMOs of the symmetric triazines are degenerated due to their symmetric molecular structures. The electron density distributions of the triazines also support the above analysis, where the HOMOs are delocalized on the donor moieties and the LUMOs are on the acceptor moieties (Fig. S4 and S5). The asymmetric triazines tend to produce asymmetric distributions of electron density, leading to clearly separated HOMOs and LUMOs. This distinct difference of the frontier orbital distributions between symmetric and asymmetric triazines should be the main reason for their distinct difference in Δ*E*_ST_.

### HOMO-LUMO overlap extent

**Figure 3:**
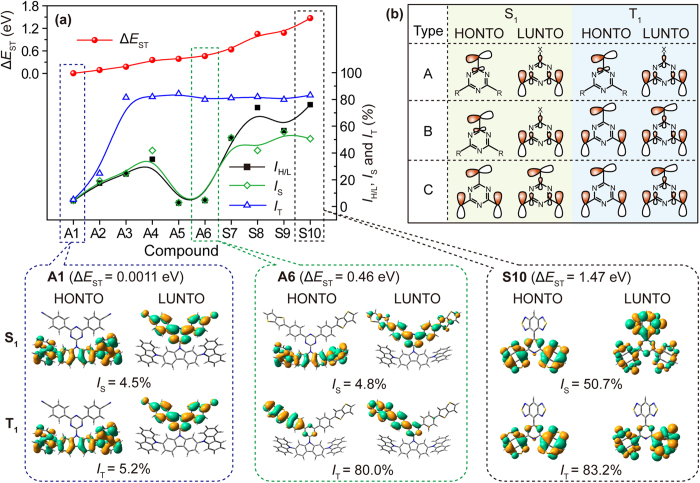
The influence of the frontier orbital overlap on Δ*E*_ST_. (**a**) The relations between the calculated Δ*E*_ST_, *I*_H/L_, *I*_S_ and *I*_T_ of triazines. Insets: HONTO and LUNTO for S_1_ and T_1_ of **A1**, **A6**, and **S10** from left to right, respectively; (**b**) The types of triazines with different HONTO-LUNTO overlap patterns at S_1_ and T_1_ states.

To give a quantitative investigation of HOMO-LUMO overlap, their overlap extent (*I*_H/L_) was calculated using Multiwfn^24^. As illustrated in Fig. 3a, Δ*E*_ST_ gradually increases from **A1** to **S10**, when molecular symmetry changes from asymmetric to symmetric with increasing *I*_H/L_. However, in the cases of compounds **A5** and **A6**, despite their obvious HOMO-LUMO separation with low *I*_H/L_ and comparable average HOMO-LUMO separation distance (<*r*_H/L_>) to that of **A1** which has an ultralow Δ*E*_ST_, they exhibit quite large Δ*E*_ST_. This is apparent contrary to the general understandings expressed in equation (1, 2, 3, 4, 5, 6, 7, 8), suggesting that there are also other undiscovered factors that influence Δ*E*_ST_ significantly.

The inconsistence between *I*_H/L_ and Δ*E*_ST_ can be also observed in TADF molecules (compound **2** in Table S1) experimentally investigated by Adachi *et al.*^11^ recently. Notably, this inconsistence may not be the calculations errors of B3LYP; other methods also well reproduced the mismatch between *I*_H/L_ and Δ*E*_ST_ (Table S2). One main reason for this mismatch is possibly that it is not accurate to use only one transition mode of HOMO → LUMO to describe the transition nature of S_1_ or T_1_ states. The TD-DFT calculations usually describe excited states in terms of various combinations of transitions between canonical molecular orbitals, and S_1_ and T_1_ are described by a set of transitions, *e.g.*, HOMO → LUMO, HOMO → LUMO + 1, etc^31^. Thus, a simple consideration of HOMO → LUMO transition may overlook intrinsic photophysical essence, leading to false estimations of optoelectronic properties and Δ*E*_ST_, especially when the content of HOMO → LUMO transition is low or symmetrically forbidden. From Tables S1 and S3, the HOMO → LUMO transition was absent in the compositions of T_1_ of compounds **2**, **A5** and **A6**, leading to obviously mismatched *I*_H/L_ and Δ*E*_ST_.

### The HONTO-LUNTO overlap extent

To consider a whole picture of electron transitions in excited states, natural transition orbital (NTO) analysis, obtained *via* the singular value decomposition of the 1-particle transition density matrix (T), was performed to offer a compact orbital representation for the electronic transition density matrix^31^,^32^. All one electron properties associated with the transition can be interpreted in a transparent way as a sum over the occupied natural transition orbitals, each orbital being paired with a single unoccupied orbital, weighted with the appropriate eigenvalue, providing a convenient description of an excited state with fewer orbital pairs than the ones given on the basis of frontier molecular orbitals. The overlap extents between HONTO and LUNTO at S_1_ (*I*_S_) and T_1_ (*I*_T_) states, which take full considerations of electron transition components at the corresponding excited states, were also calculated using Multiwfn^24^. From Fig. 3a, symmetric triazines generally have high *I*_S_ and *I*_T_, but high *I*_T_ can be also observed in asymmetric molecules, leading to relatively large Δ*E*_ST_ of those compounds^33^. Typically, in compounds of **A5** and **A6** that were misunderstood by low *I*_H/L_, their *I*_S_ are very low (<5%), but the *I*_T_ are around 80%, suggesting that there are severe overlaps at T_1_ and the high *I*_T_ should be very likely the main reason for their relatively large Δ*E*_ST_ (~0.45 eV).

Take a close look at the HONTO and LUNTO distributions at S_1_ and T_1_. When Δ*E*_ST_ is extremely low (0.0011 eV) as in **A1**, both HONTO and LUNTO are separated with low *I*_S_ and *I*_T_ (~5%); when Δ*E*_ST_ is relatively high (0.46 eV) as in **A6**, HONTO and LUNTO are separated at S_1_ with low *I*_S_ (<5%) but they are overlapped at T_1_ with high *I*_T_ (~80%); when HONTO and LUNTO are overlapped at both S_1_ and T_1_ with high *I*_S_ and *I*_T_ (~50% and 80% respectively) as in **S10**, very high Δ*E*_ST_ (1.47 eV) can be resulted. To this end, three types of molecules can be distinguished according to HONTO and LUNTO overlap pattern (Fig. 3b). In **Type A**, ultralow Δ*E*_ST_ is resulted from the separated HONTO and LUNTO at both S_1_ and T_1_ (small *I*_S_ and small *I*_T_) states. In **Type B**, moderately low Δ*E*_ST_ can be observed with separated HONTO and LUNTO at S_1_ state but overlapped HONTO and LUNTO at T_1_ (small *I*_S_ but large *I*_T_) state. **Type C** has large Δ*E*_ST_ due to overlapped HONTO and LUNTO at both S_1_ and T_1_ states (large *I*_S_ and large *I*_T_). The newly revealed relation between Δ*E*_ST_ and overlap extents of *I*_S_ and *I*_T_ highlights the importance of full consideration of molecular orbital participations at related spin states, when studying the exciton transfer processes between these excited states. Notably, these finds are independent of TD-DFT computational functionals; as presented in Table 1, same relations can be also concluded from other computational methods.

### The frontier orbital separation distance

The success in dividing molecules into **Types A**, **B**, and **C** according to *I*_S_ and *I*_T_ qualitatively cannot be achieved when analyzing their individual difference quantitatively. For example, **S7** has higher *I*_S_ and similar *I*_T_ in comparison with **S8**, but its Δ*E*_ST_ is quite lower than that of **S8**. The same inconsistence can be also found between **A4** and **A5**. From equation (3), Δ*E*_ST_ was determined not only by the molecular orbital overlap but also by their separation distance^34^. Larger separation distance leads to lower Δ*E*_ST_^35^. Hence, we need to further quantitatively investigate the effects of mean separation distances between HOMO and LUMO (<*r*_H/L_>), and between HONTO and LUNTO at S_1_ (<*r*_S_>) and T_1_ (<*r*_T_>).

**Figure 4:**
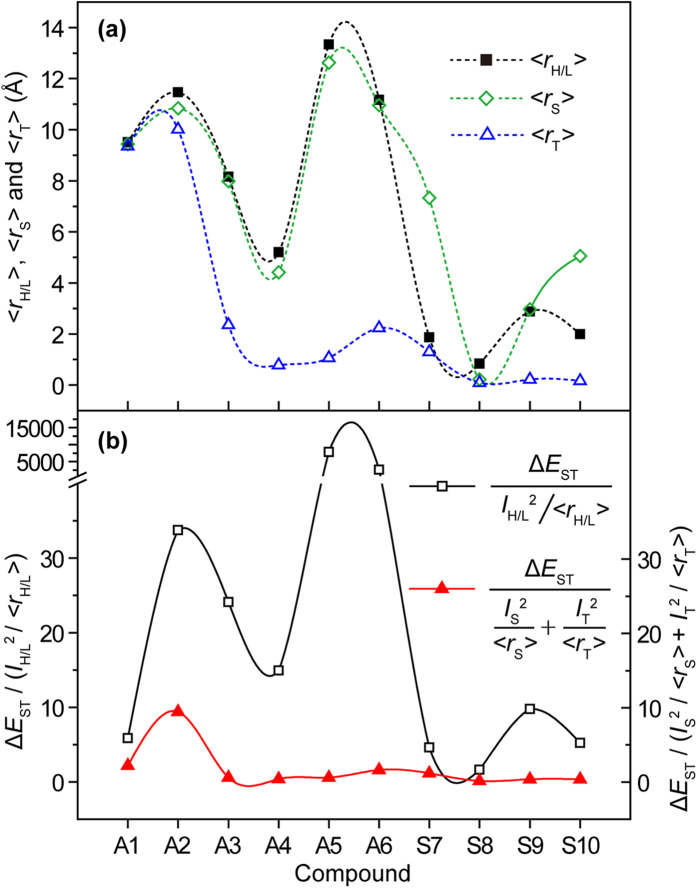
The influence of the frontier orbital separation distance on Δ*E*_ST_. (**a**) The <*r*_H/L_>, <*r*_S_> and <*r*_T_>. (**b**) The values of Δ*E*_ST_/(*I*_H/L_^2^/<*r*_H/L_>) and Δ*E*_ST_/(*I*_S_^2^/<*r*_S_> + *I*_T_^2^/<*r*_T_>).

From **A1** to **S10** whose Δ*E*_ST_ increases gradually, the expected gradually decreased <*r*_H/L_> was broken obviously by **A5** and **A6**; **A5** has the largest <*r*_H/L_> (Fig. 4a). This abnormal is in line with their unexpectedly low *I*_H/L_ in Fig. 3a, indicating again the inaccuracy of *I*_H/L_ and <*r*_H/L_> in assessing Δ*E*_ST_. Benefited from NTO analysis on the whole picture of the electron transitions for the excited states, the low <*r*_T_> of **A5** and **A6** should be a main reason for their high Δ*E*_ST_, although their <*r*_S_> are also quite high. Notably, symmetric triazines generally have lower <*r*_H/L_>, <*r*_S_>, and <*r*_T_> than asymmetric triazines, resulting in the high Δ*E*_ST_ according to equation (3).

To elucidate quantitative relations between Δ*E*_ST_ and factors of *I*_H/L_ and <*r*_H/L_>, we simplified equation (3) to get equation (9) (see Supplementary Information).


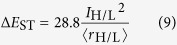


where Δ*E*_ST_ is in eV and <*r*_H/L_> is in Å. From the 35 compounds except for **A5** and **A6**, the average Δ*E*_ST_/(*I*_H/L_^2^/<*r*_H/L_>) is 25.7 (Fig. S6)^36^, which is very close to the 28.8 in equation (9). The very high values observed in **A5** and **A6** (Fig. 4b) suggest again the unfitness of the normal HOMO-LUMO transition analysis, since there are very low HOMO → LUMO transition components for their T_1_ states. Therefore, it is necessarily to address not only the conventional HOMO → LUMO transition but also the other frontier orbital transition components.

With the aid of NTO analysis to contain all the possible transitions, new parameters of *I*_S_, *I*_T_, <*r*_S_>, and <*r*_T_> were obtained and found to be useful in investigating the influence factors of Δ*E*_ST_. The quantitative relations were supposed to be a linear combination of S_1_ and T_1_ components in equation (10):


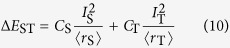


where *C*_S_ and *C*_T_ are combination constants of S_1_ and T_1_ states, respectively. From all the compounds studied (**A1** **~** **S37**) including **A5** and **A6**, the nonlinear least square fitted *C*_S_ and *C*_T_ are found to be 0.23 and 0.39 respectively with R-square of 0.9685. If *C*_S_ is set to be equal to *C*_T_, they were changed to 0.385 with R-square of 0.9912. The higher *C*_T_ than *C*_S_ in the first fit with slight decrease in the second fit, suggests that the T_1_ component plays a dominate role in determining Δ*E*_ST_. For all the 37 studied compounds, Δ*E*_ST_/(*I*_S_^2^/<*r*_S_> + *I*_T_^2^/<*r*_T_>) varies in a relatively narrow range from 0.15 to 11.16 (Fig. 4b and S6), indicating that the simplified equation (10) presents a good correlation between Δ*E*_ST_ and parameters of *I*_S_, *I*_T_, <*r*_S_>, and <*r*_T_> derived from NTO analysis. Thus, it is advisable to consider the whole picture of frontier orbital transitions (HONTO and LUNTO) at both S_1_ and T_1_ states to accurately understand Δ*E*_ST_ tuning.

### Design of ideal TADF molecules with ultralow Δ*E*_ST_

In light of the sophisticated tuning of Δ*E*_ST_ *via* symmetric control on *I*_S_ and *I*_T_ and distance control on <*r*_S_> and <*r*_T_>, we first use the above developed molecular design strategy to construct high-performance TADF molecules with ultralow Δ*E*_ST_; the development of novel TADF molecules is one of the hottest topics in current research of organic electronics^8^. To test the validity of the above studies on the relations between Δ*E*_ST_ and these new parameters of *I*_H/L_, *I*_S_, *I*_T_, <*r*_H/L_>, <*r*_S_> and <*r*_T_>, four efficient TADF molecules recently reported by Adachi and co-workers^11^ was investigated. Indeed, the smaller *I*_H/L_ cannot lead to lower Δ*E*_ST_; the larger Δ*E*_ST_ can be explained by the larger *I*_T_ of these molecules (Table S1); the higher *I*_S_ with higher frontier orbital overlap at S_1_ state may lead to higher luminescent efficiency of the D-A molecules when the low *I*_T_ can still maintain a small Δ*E*_ST_. Still, these finds are independent of calculation methods (Table S2). Our approach gave a good prediction on the reported experimental results, indicating the high reliability of these new parameters for Δ*E*_ST_ describing.

**Figure 5:**
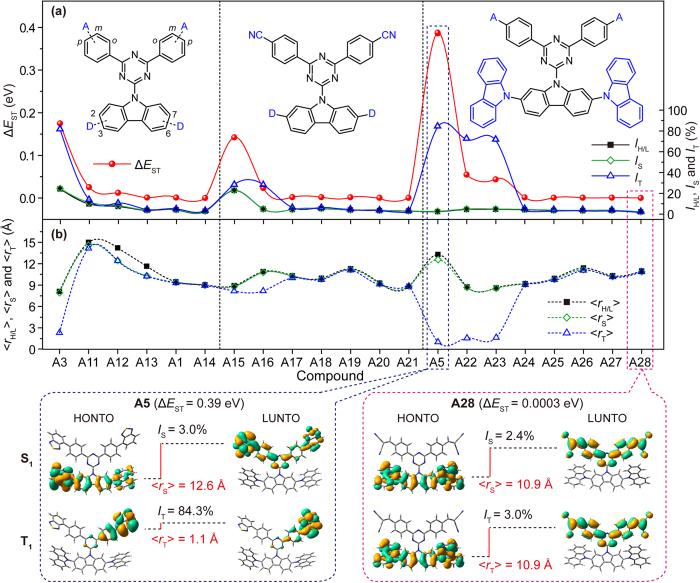
The Δ*E*_ST_ of the designed asymmetric triazines TADF molecules. (**a**) The overlap extents of *I*_H/L_, *I*_S_ and *I*_T_ and (**b**) the average frontier orbital separation distances of <*r*_H/L_>, <*r*_S_> and <*r*_T_>. Insets: HONTO and LUNTO for S_1_ and T_1_ of **A5** (left) and **A28** (right).

Based on triazine architecture, we adopt an asymmetric molecular structure to minimize Δ*E*_ST_ by fine-tuning the substitution positions and varied types of donor and acceptor substituents^37^. Started from the asymmetric triazine molecule of **A3**, which is an efficient host material for phosphorescent OLED with experimental Δ*E*_ST_ of 0.34 eV^27^, we enhanced the electron donating ability of the carbazolyl substituent by introducing additional donors of carbazole at 2,7- or 3,6- position; on the other hand, we enhanced the electron accepting ability of the two phenyl substituents by attaching the strong acceptor of cyano group (CN) at the *para* (*p*), *meta* (*m*), or *ortho* (*o*) positions (Fig. 5a). According to the TD-DFT calculations, this strategy is succeed in producing ultralow (almost zero) Δ*E*_ST_ especially in **A1**, **A13**, and **A14**, when the additional donors are connected through 2,7-positions of the carbazolyl substituent and the CN is introduced either at *p*, *m*, or *o* position. The 3,6- connection results in slight HOMO distribution on triazine core, which will overlap with LUMO distribution, leading to slightly higher Δ*E*_ST_ of **A11** (Table S4 and Fig. S5a). Besides carbazolyl substituents, other electron donating groups such as alkyl, phenyl, diamine, alkoxyl, etc. as in **A16** **~** **A21,** are also effective in reducing Δ*E*_ST_ (Fig. S5b). But, without the additional donors to increase the HOMO, Δ*E*_ST_ will be apparently increased as found in **A15**. Also, without the additional acceptors on the phenyl substituents to reduce the LUMO, Δ*E*_ST_ will be large as in **A22**. Still, other kinds of accepting groups of trifluoromethyl, diphenylphosphoryl, nitryl, diphenylboronyl, and 2-methylenemalononitrile in **A24** **~** **A28**, work well too, producing ultralow Δ*E*_ST_ (low to 0.0003 eV in **A28**) (Fig. S5c). However, fluoro and benzothiazolyl substitution cannot lead to low Δ*E*_ST_ due to their failure in avoiding overlap at both S_1_ and T_1_; the large *I*_T_ and small <*r*_T_> of **A5** and **A23** clearly indicates the large overlap at T_1_ states (Fig. 5a,b). Compared to the experimentally investigated TADF molecule of **A2**, which has exhibit an external quantum efficiency (EQE) of 14% ± 1% with the experimental Δ*E*_ST_ of 0.02 eV and calculated one of 0.09 eV based on B3LYP/6-31G(d) (Table 1)^28^, these newly designed TADF molecules are expected to have improved device performance, considering their well separated S_1_ and T_1_ with low *I*_S_ and *I*_T_ and long <*r*_S_> and <*r*_T_> simultaneously.

### Design of TTA and SF molecules with large Δ*E*_ST_

Contrary to the asymmetric triazine-based TADF molecules showing very low Δ*E*_ST_, symmetric triazines can lead to large Δ*E*_ST_ which is required for TTA and SF molecules. According to equation (9) and (10), large Δ*E*_ST_ needs large *I*_H/L_, *I*_S_, and *I*_T_ as well as short <*r*_H/L_>, <*r*_S_>, and <*r*_T_>. In other words, large transition orbital overlap with localized excitation will result in large Δ*E*_ST_^34^. Here, two approaches were adopted to design symmetric triazine-based TTA and SF molecules. The first one is to use electron-withdrawing substituents to make the S_1_ and T_1_ locally excited. The other one is by introducing polycyclic aromatic fragments, which have been widely used in many TTA and SF molecules due to their large conjugation beneficial for electron localization at excited states.

**Figure 6:**
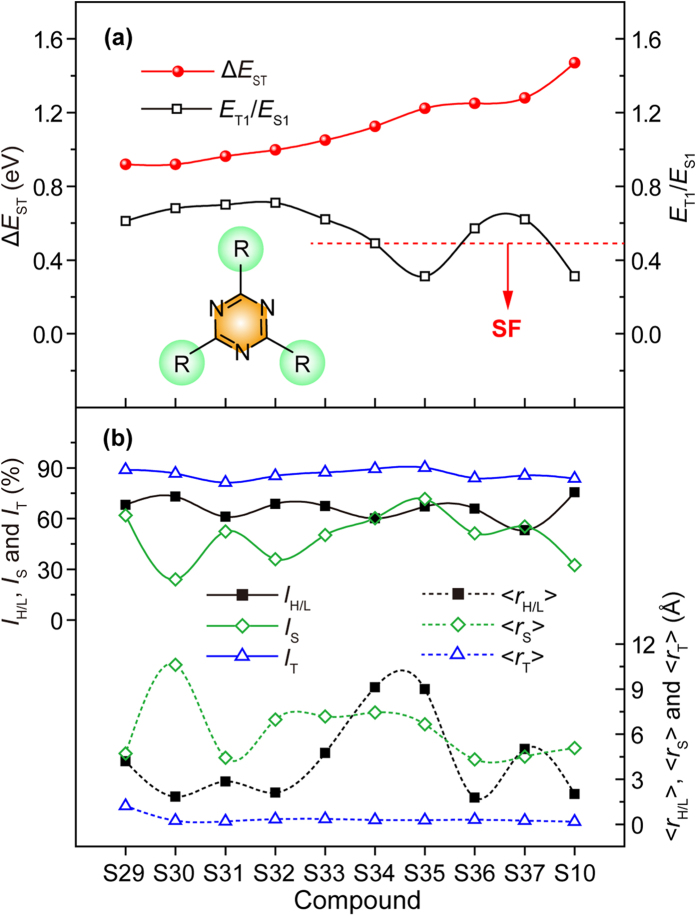
The designed symmetric triazines for TTA and SF. (**a**) Δ*E*_ST_ and *E*_T1_/*E*_S1_; (**b**) *I*_H/L_, *I*_S_, *I*_T_, <*r*_H/L_>, <*r*_S_> and <*r*_T_>.

In Fig. 6a, the molecules are arranged in an increasing order of Δ*E*_ST_ from 0.92 to 1.47 eV. When *E*_T1_/*E*_S1_ is close but higher than 0.5, TTA process is supposed potentially to be applicable; when *E*_T1_/*E*_S1_ is lower than 0.5, SF process is possible^18^,^19^. According to these criterions, **S10**, **S34**, and **S35** are SF molecules, while **S29** **~** **S33**, **S36** and **S37** are TTA molecules. From Fig. S7, the electron acceptor of triazine core participates the formation of LUMO to a large content for all the symmetric compounds, while it shows only apparent effects on HOMOs of **S36** and **S37** whose substituents are acceptors, because HOMO is dominated by the donor unit in D-A molecules. Delocalized HONTO and LUNTO at both S_1_ and T_1_ of **S36** was also observed, which is in contradictory to the D-A molecule of **S35** (Fig. S8a and b). The more localized and overlapped HONTO and LUNTO of **S35** makes it a good SF molecule with lower *E*_T1_ and *E*_T1_/*E*_S1_. Notably, the increase of Δ*E*_ST_ will not certainly leads to SF molecules; the T_1_ energy of **S36** and **S37** are comparably too high (Table S5), resulting in *E*_T1_/*E*_S1_ > 0.5, although their Δ*E*_ST_ are among the largest ones. The high-lying T_1_ may have close relations with the triazine core^38^. The large participation of the triazine core at T_1_ of **S36** was further confirmed by its delocalized spin density distribution (Fig. S8c). Since the triazine core has high T_1_, its large participation may enable the compound to inherit the high T_1_ of triazine, resulting in high *E*_T1_/*E*_S1_ of **S36** and **S37**.

From Fig. 6b, significantly higher *I*_H/L_ and shorter <*r*_H/L_> of the symmetric triazines in comparison with that of asymmetric triazines were observed, demonstrating the success in modifying the molecular orbital overlap and separation distance *via* symmetry control in designing molecules with large Δ*E*_ST_ for TTA or SF processes. As further revealed by *I*_S_ and *I*_T_, heavier overlap seems to happen at T_1_ with much shorter <*r*_T_> than <*r*_S_>, highlighting the dominative role of T_1_ in the enlargement of Δ*E*_ST_. For the construction of triazine-based TTA and TADF molecules, they can be facilely designed by symmetrically introducing TTA molecules of perylene, pyrene, and anthracene or SF molecules of naphthacene and pentacene correspondingly. Typically, the S_1_ energy of **S10** with the largest Δ*E*_ST_ of 1.47 eV is more than twice higher than its T_1_ energy, affording **S10** to be a good candidate for SF process^18^.

## Discussion

We have succeed in manipulating excited state electronic structures for accommodation of various organic excitons *via* symmetry control of Δ*E*_ST_ in a wide range from ultralow (0.0003 eV) for TADF and extra-large (1.47 eV) for SF all in a triazine-based molecular architecture based on a combined quantum chemistry modeling and experimental exploring. The HOMO-LUMO overlap (*I*_H/L_) and separation distance (<*r*_H/L_>) were quantified successfully. It was found that asymmetry triazines possess separated HOMO-LUMO with low *I*_H/L_ and long <*r*_H/L_>, leading to low Δ*E*_ST_; while symmetry triazines contain highly overlapped HOMO-LUMO with high *I*_H/L_ and short <*r*_H/L_>, resulting in large Δ*E*_ST_. However, it is difficult for *I*_H/L_ and <*r*_H/L_> to well describe Δ*E*_ST_. Consequently, we further developed a set of new parameters of *I*_S_, *I*_T_, <*r*_S_>, and <*r*_T_> benefitted from NTO analysis to consider whole pictures of the electron transitions at both S_1_ and T_1_ states. According to these firstly proposed parameters, three types of molecules can be classified. **Type A** has ultralow Δ*E*_ST_ due to both separated S_1_ and T_1_ with low *I*_S_ (long <*r*_S_>) and *I*_T_ (long <*r*_T_>); **Type B** has small Δ*E*_ST_ due to separated S_1_ but overlapped T_1_ with low *I*_S_ (long <*r*_S_>) and high *I*_T_ (short <*r*_T_>); **Type C** shows large Δ*E*_ST_ due to overlapped S_1_ and T_1_ with high *I*_S_ (short <*r*_S_>) and *I*_T_ (short <*r*_T_>). A quantitative relation between Δ*E*_ST_ and *I*_S_, *I*_T_, <*r*_S_>, and <*r*_T_> was established and the T_1_ component was found to play a dominate role in determining Δ*E*_ST_. These findings are important in providing quantitative approaches for fundamental understandings on the intrinsic factors influencing Δ*E*_ST_ tuning, representing a major step towards technological advances in expanding the scope of excited state manipulation.

## Methods

The molecular geometries in the ground state (S_0_) were optimized *via* spin-restricted DFT calculations at the B3LYP/6-31G(d) level of theory using Gaussian 09 package^39^. The spin-unrestricted formalism was used in geometry optimization of the lowest triplet excited state (T_1_). Vibrational frequency calculations were subsequently carried out to confirm that all the optimized structures are corresponding to the minima on the potential energy surfaces. The excited singlet (S_n_) and triplet (T_n_) states were investigated by the time-dependent DFT (TD-DFT) formalism with the same functional and basis set of B3LYP/6-31G(d) on the optimized ground-state geometries^40^. TD-DFT calculations based on the standard B3LYP functional offer a reasonable description for singlet and triplet states of medium-sized molecules, which has been widely used in the theoretical studies of TADF, TTA and SF molecules^11^,^41^. To obtain a precise picture of the excited states, we further performed natural transition orbitals (NTOs) analysis, which can offer a compact orbital representation for the electronic transition density matrix^36^.

To get a solid support of the computational study, the experimental measurements of the synthesized triazines were analyzed and compared. The detailed synthesis, structure characterizations, and photophysical property measurements of these triazines can be found in Supplementary Information.

## Additional Information

**How to cite this article**: Chen, T. *et al.* Understanding the Control of Singlet-Triplet Splitting for Organic Exciton Manipulating: A Combined Theoretical and Experimental Approach. *Sci. Rep.* **5**, 10923; doi: 10.1038/srep10923 (2015).

## Supplementary Material

Supplementary Information

## Figures and Tables

**Figure 1:**
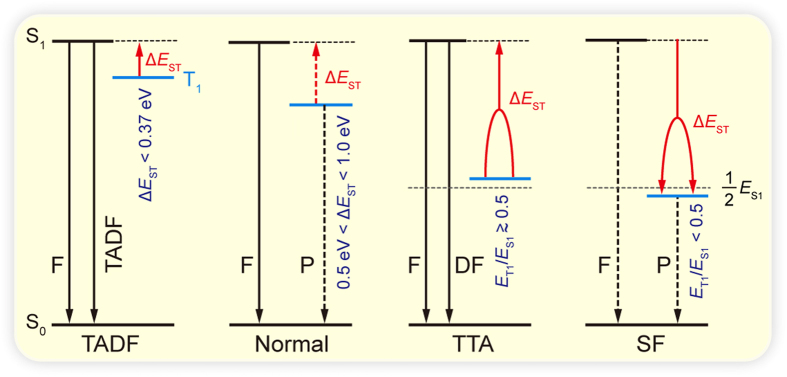
Energy level diagrams depicting diversified photophysical processes determined by singlet-triplet splitting (Δ*E*_ST_) between energies of the lowest singlet (*E*_S1_) and triplet (*E*_T1_) excited states. Noted that F, P, DF, TADF, TTA, SF represent fluorescence, phosphorescence, delayed fluorescence, thermally activated delayed fluorescence, triplet-triplet annihilation, and singlet fission, respectively. The weak emissions and transitions are in dotted line.

**Table 1:** Calculated Δ*E*_ST_ (eV) and frontier orbital overlap extents (*I*_H/L_, *I*_S_, and *I*_T_ (%)) using various functionals at 6–31G(d) basis set level in comparison with experimental data of Δ*E*_ST_ (eV).

**Compound**	**A2**		**A3**		**A4**		**S9**		**A15**	
	**△*****E***_**ST**_	***I***_**H/L**_**/*****I***_**S**_**/*****I***_**T**_	**△*****E***_**ST**_	***I***_**H/L**_**/*****I***_**S**_**/*****I***_**T**_	**△*****E***_**ST**_	***I***_**H/L**_**/*****I***_**S**_**/*****I***_**T**_	**△*****E***_**ST**_	***I***_**H/L**_**/*****I***_**S**_**/*****I***_**T**_	**△*****E***_**ST**_	***I***_**H/L**_**/*****I***_**S**_**/*****I***_**T**_
B3LYP	0.09	17/19/25	0.17	24/25/81	0.36	35/42/82	1.08	57/56/80	0.14	23/23/29
PBE0	0.34	17/20/85	0.46	24/26/83	0.64	35/43/83	1.32	56/56/80	0.22	23/24/83
BMK	0.54	17/23/85	0.56	24/30/81	0.67	34/46/83	1.46	56/58/80	0.37	22/26/83
M062X	0.63	17/27/85	0.60	24/34/81	0.59	34/49/83	1.41	55/64/79	0.44	22/29/83
M06HF	0.66	17/72/71	0.84	23/65/81	0.66	34/54/77	1.35	55/69/78	0.90	21/49/83
ωB97XD	1.15	16/70/85	1.23	23/38/84	1.20	34/51/84	1.78	55/69/80	1.08	21/33/84
CAM-B3LYP	1.27	16/69/86	1.27	23/35/85	1.30	34/49/84	1.89	56/69/81	1.07	22/30/84
Exp.	0.02^26^	—	0.34^25^	—	0.45^25^	—	1.01	—	0.19	—
