# Supplementary material for: Understanding the Control of Singlet-Triplet Splitting for Organic Exciton Manipulating: A Combined Theoretical and Experimental Approach
Source: Sci Rep. 2015 Jul 10;5:10923. doi: 10.1038/srep10923 (PMC4498327; doi:10.1038/srep10923)
Supplement: Supplementary Information [file srep10923-s1.pdf]

Supplementary Information for

Understanding the Control of Singlet-Triplet Splitting for Organic Exciton Manipulating: A Combined Theoretical and Experimental Approach

Ting Chen[1], Lei Zheng[1], Jie Yuan[1], Zhongfu An[1,2], Runfeng Chen[1]*, Ye Tao[1], Huanhuan Li[1], Xiaoji Xie[2], & Wei Huang[1,2]*

[1]*Key Laboratory for Organic Electronics and Information Displays & Institute of Advanced Materials, Jiangsu National Synergistic Innovation Center for Advanced Materials, Nanjing University of Posts & Telecommunications, 9 Wenyuan Road, Nanjing 210023, China*

[2]*Key Laboratory of Flexible Electronics & Institute of Advanced Materials, Jiangsu National Synergistic Innovation Center for Advanced Materials, Nanjing Tech University, 30 South Puzhu Road, Nanjing 211816, China*

Contents

1. **Synthesis of organic molecules**
2. **Photophysical property measurements**
3. **The singlet-triplet splitting (ΔE_{ST})**
   - **3.1 The calculation of overlap extent**
   - **3.2 The calculation of molecular orbital separation distance**
   - **3.3 Natural transition orbital (NTO) analysis**
4. **Supplementary figures**
5. **Supplementary tables**

References

1. Synthesis of organic molecules

Materials. Unless otherwise noted, all reactions were carried out under a nitrogen atmosphere using standard Schlenk techniques. Tetrahydrofuran (THF) was dried and distilled over sodium/benzophenone. All other chemicals and deuterated solvents were obtained from commercial sources and used as received unless otherwise noted. The detailed synthesis and molecular structure characterization of the intermediate product of 2-Carbazolyl-4,6-dichloro-1,3,5-triazine can be found in previous reports.[1,2]

Molecular structure characterization. ^{1}H NMR and ^{13}C NMR spectra were recorded on a Bruker Ultra Shield Plus 400 MHz spectrometer using tetramethylsilane (TMS) as the internal standard. GC-MS experiments were performed on a Shimadzu GCMS-QP2010.

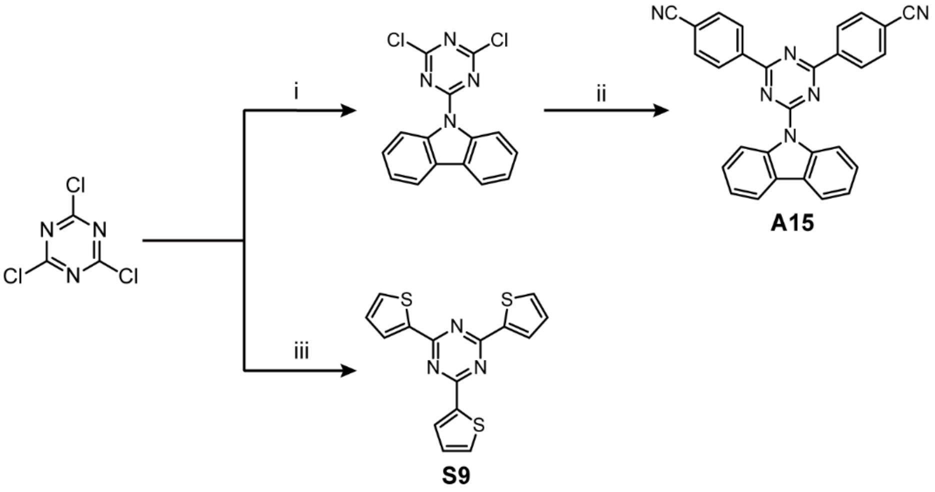

Scheme S1. Synthetic routes of triazine derivatives of **A3** and **S9**. (i) 1.1 *equiv.* *n*-BuLi, carbazole, 0 °C, 2 h; (ii) 2.2 *equiv.* 4-cyanophenylboronic acid, $Pd(PPh_3)_4$, 85 °C, 48 h; (iii) 3.2 *equiv.* Mg, 2-bromothiophene, 85 °C.

9-(4,6-dichloro-1,3,5-triazin-2-yl)-9H-carbazole. To a carbazole (1.0 g; 6.0 mmol) dry THF (10 ml) solution at 0 °C was added 2.5 M *n*-butyllithium (2.6 ml, 6.5 mmol). The mixture was stirred at 0 °C for 2 h, then 5 ml methanol was added into to quench the reaction. The organic solvents were removed *in vacuo*. The residue was washed using acetone for 3 times to afford a white powder. Yield: 1.70 g: (90%).

4,4'-(6-(9H-carbazol-9-yl)-1,3,5-triazine-2,4-diyl)dibenzonitrile (A15). 2-Carbazolyl-4,6-dichloro-1,3,5-triazine (0.5 g; 1.6 mmol), 4-cyanophenylboronic acid (0.51 g; 34.7 mmol), aliquat336, and 2.0 mol% $Pd(PPh_3)_4$ were dissolved in a mixture of anaerobic toluene (20 mL) and potassium carbonate aqueous solution (8 mL, 2 M) under the protection of nitrogen atmosphere. The reaction mixture was

vigorously stirred at 90 °C for 48 h, then was quenched by pouring into deionized water (100 mL) and extracted by dichloromethane (3×100 mL). The organic layer was separated, collected, and dried over anhydrous Na_2SO_4. The solvent was removed *in vacuo*, and the residue was purified by flash column chromatography (eluent: petroleum ether/ethyl acetate = 4/1) to afford **A15** (0.46 g; Yield: 65%) as a yellow powder. 1H NMR ($CDCl_3$, 400 MHz, ppm): 9.038 (d, 2H), 8.834 (d, 4H), 8.114 (d, 2H), 7.944 (d, 4H),7 .623 (t, 2H),7.489(t, 2H). ^{13}C NMR (100 MHz, $CDCl_3$, ppm): 171.224, 165.151, 139.830, 138.822, 132.700, 129.545, 127.280, 127.027, 124.069, 119.983, 118.271, 117.602, 116.262. GC-MS (m/z): calcd for $C_{29}H_{16}N_6$: 448.14, found: 448.

2,4,6-tri(thiophen-2-yl)-1,3,5-triazine (S9). The Griganard reagent of thiophenylmagnesium bromide in THF (50 ml) prepared from magnesium (0.108 g; 4.5 mmol) and 2-bromothiophene (0.708 g; 4.4 mmol) was added dropwise into a THF solution (20 ml) of 2,4,6-trichloro-1,3,5-triazine (0.2 g; 1.1 mmol). The mixture was allowed to be stirred at 80 °C for 24 h, then was poured into deionized water (100 ml) to quench the reaction and extracted by dichloromethane (3×100 ml). The organic layer was separated and dried over anhydrous magnesium sulfate. The solvent was removed under reduced pressure, and the residue was further purified by column chromatography on silica gel using a mixed eluent (petroleum ether: ethyl acetate = 15:1). Yield: 0.32 g of aquamarine powder (70%). ^1H NMR (400 MHz, $CDCl_3$, ppm): 8.28 (d, 3H), 7.62 (d, 3H), 7.22 (t, 3H). ^{13}C NMR (100 MHz, $CDCl_3$, ppm): 167.74, 141.44, 132.37, 131.73, 128.47. GC-MS (m/z): calcd for $C_{15}H_9N_3S_3$: 327.45, found: 327.

2. Photophysical property measurements

UV/Vis absorption spectra were recorded on a PerkinEImer UV/Vis Spectrometer Lambda 35. Fluorescence spectra were obtained using a PerkinEImer LS 55 Fluorescence Spectrometer. The phosphorescence spectra of the compound (in chloroform) were measured using an Edinburgh LFS920 fluorescence spectrophotometer at 77 K with a 5 ms delay time after excitation using a microsecond flash lamp.

The experimental E_{S1} of the compound was determined from the crossing point of the normalized absorption and emission spectra in chloroform at room temperature.[3] The experimental E_{T1} of the compound is determined from the highest energy vibronic component of its phosphorescence spectrum at 77 K.[4]

3. The singlet-triplet splitting (ΔE_{ST})

In principle, the molecular energy at the lowest singlet (E_{S1}) or triplet (E_{T1}) excited states is decided by the sum of the orbital energy (E), electron repulsion energy (K) and electron exchange energy (J) of the two unpaired electrons on the frontier orbitals (φ_H and φ_L). The electrons at the same molecular orbital have the same E, K and J, however, the spin paired electrons have a positive J while the spin unpaired electrons have a negative J.[5] As described in equations (S1)-(S3), the exchange energy J is the most decisive factor for the singlet-triplet energy splitting of ΔE_{ST}.

$$E_{S1} = E + K + J \tag{S1}$$

$$E_{T1} = E + K - J \tag{S2}$$

$$\Delta E_{ST} = E_{S1} - E_{T1} = 2J \tag{S3}$$

$$J = \iint \varphi_L(1)\varphi_H(1)\left(\frac{e^2}{r_1 - r_2}\right)\varphi_L(2)\varphi_H(2)\, dr_1\, dr_2 \tag{S4}$$

$$\Delta E_{ST} = \iint \varphi_L(1)\varphi_H(1)\left(\frac{2e^2}{r_1 - r_2}\right)\varphi_L(2)\varphi_H(2)\, dr_1\, dr_2 \tag{S5}$$

From equation (S4), J is determined by spatial separation (r_1-r_2) and overlap integral of φ_H and φ_L, *i.e.*, spatial wave function separation of frontier orbitals.[6] Thus a small ΔE_{ST} can be expected when there is a small overlap or a large separation between HOMO and LUMO, and a large overlap or a small separation will lead to a large ΔE_{ST}.[7] Their relations were demonstrated in Scheme S2.

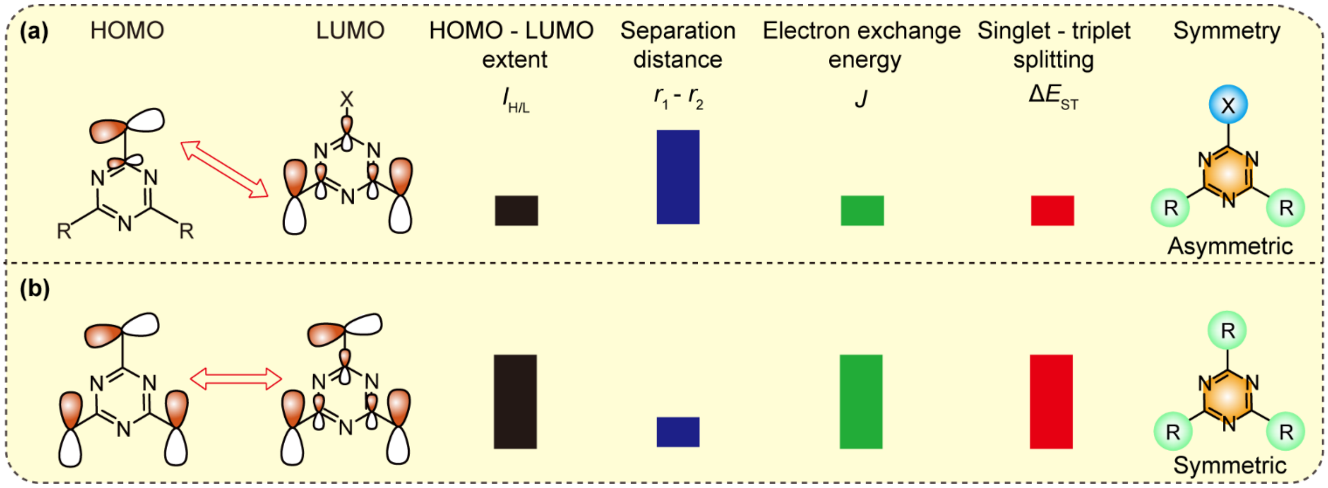

Scheme S2. The relations between the singlet-triplet splitting (ΔE_{ST}), HOMO-LUMO overlap, and molecular symmetry demonstrated in **(a)** asymmetric and **(b)** symmetric triazines.

3.1 The calculation of overlap extent

Using the overlap integral function embedded in Multiwfn,[8] the overlap between two orbitals (φ_i and φ_j) of a molecule can be calculated as in equation (S6).

$$I = \int |\varphi_i(r)||\varphi_j(r)| dr \quad \text{(S6)}$$

Thus, the extent of HOMO (φ_H) and LUMO (φ_L) overlap is

$$I_{\mathrm{H/L}} = \int |\varphi_H(r)||\varphi_L(r)| dr \quad \text{(S7)}$$

More details about the orbital overlap calculations can be found in Multiwfn manual.[9]

3.2 The calculation of the molecular orbital separation distance

Adopting the function outputting statistic data of the points in specific spatial and value range, the barycenter (r_{tot}) of the absolute value of the molecular orbital can be computed as in equation (S8).

$$r_{\mathrm{tot}} = \sum_k^{all} r_k f(r_k) / \sum_k^{all} f(r_k) \quad \text{(S8)}$$

where f is the data value, r denotes coordinate vector, k runs over all grid points including positive and negative points respectively. Thus, the barycenter of HOMO and LUMO (r_{H} and r_{L} respectively) are

$$r_{\mathrm{H}} = \sum_k^{all} r_k f_H(r_k) / \sum_k^{all} f_H(r_k) \quad \text{(S9)}$$

$$r_{\mathrm{L}} = \sum_k^{all} r_k f_L(r_k) / \sum_k^{all} f_L(r_k) \quad \text{(S10)}$$

And the mean distance between HOMO and LUMO is

$$< r_{\mathrm{H/L}} > = |r_{\mathrm{H}} - r_{\mathrm{L}}| \quad \text{(S11)}$$

Supposing that the separation distance between HOMO and LUMO (r_1-r_2) is varying in a small range around ($<r_{\mathrm{H/L}}>$), equation (S5) can be simplified using equation (S11) to get equation (S12).

$$\Delta E_{\mathrm{ST}} = \iint \varphi_L(1)\varphi_H(1)\left(\frac{2e^2}{< r_{H/L} >}\right)\varphi_L(2)\varphi_H(2)\, dr_1\, dr_2$$

$$= \left(\frac{2e^2}{< r_{H/L} >}\right)\int \varphi_L(2)\varphi_H(2)\left[\int \varphi_L(1)\varphi_H(1) dr_1\right] dr_2$$

$$= \frac{2e^2}{< r_{\mathrm{H/L}} >} I_{\mathrm{H/L}}{}^2 \qquad \text{(S12)}$$

When ΔE_{ST} is in eV, $<r_{\mathrm{H/L}}>$ is in Å, equation (S12) can be transformed to

$$\Delta E_{\mathrm{ST}} = 28.8 \frac{I_{\mathrm{H/L}}{}^2}{< r_{\mathrm{H/L}} >} \qquad \text{(S13)}$$

However, TD-DFT calculations usually describe excited states in terms of various combinations of transitions between canonical molecular orbitals, and the first singlet excited state (S_1) and triplet excited state (T_1) are described by a set of different transitions, *e.g.*, HOMO → LUMO, HOMO → LUMO+1, etc.[10] This means that according to TD-DFT calculations, it is not accurate to use the only transition mode of HOMO → LUMO to describe the transition nature of S_1 or T_1 states. Thus, a simple picture of the excited states is often evasive.

3.3 Natural transition orbital (NTO) analysis

Natural transition orbitals (NTOs), obtained *via* the singular value decomposition of the 1-particle transition density matrix (T), can offer a compact orbital representation for the electronic transition density matrix. The transition density matrix is diagonal with a dimension of $N_{\mathrm{occ.}} \times N_{\mathrm{virt.}}$ (N_{occ}, number of occupied orbitals; N_{virt}, number of virtual orbitals):

$$\left| \left[\mathrm{U}^{\dagger}\mathrm{TV} \right]_{ij} \right| = \lambda_i \delta_{ij} \qquad \text{(S14)}$$

Here, U and V are square unitary transformation matrices of dimensions $N_{\mathrm{occ.}} \times N_{\mathrm{occ.}}$ and $N_{\mathrm{virt.}} \times N_{\mathrm{virt.}}$, respectively, and $U^{\dagger}$ denotes the conjugate transpose of matrix U; λ_i represents the singular value of matrix T; δ_{ij} is the Kronecker delta. Notably, all one electron properties associated with the transition can be interpreted in a transparent way as a sum over the occupied natural transition orbitals, each orbital being paired with a single unoccupied orbital, weighted with the appropriate eigenvalue λ_i. Hence, a NTO analysis is very convenient in providing a better description of an excited state with fewer orbital pairs than the ones given on the basis of frontier molecular orbitals.[11]

Consequently, according to equation (S7), the overlap extent of the highest occupied NTO (HONTO) ($\varphi_{\mathrm{H'}}$) and the lowest unoccupied NTO (LUNTO) ($\varphi_{\mathrm{L'}}$) at S_1 or T_1 states described by NTO analysis can be calculated in equations (S15)~(S16), respectively.

$$I_{\mathrm{S}} = \int \left| \varphi_{\mathrm{H'}}^{\mathrm{S}}(r) \right| \left| \varphi_{\mathrm{L'}}^{\mathrm{S}}(r) \right| dr \qquad \text{(S15)}$$

$$I_{\mathrm{T}} = \int \left|\varphi_{\mathrm{H}'}^{\mathrm{T}}(r)\right|\left|\varphi_{\mathrm{L}'}^{\mathrm{T}}(r)\right| dr \tag{S16}$$

And according to equations (S9) and (S10), the barycenters of HONTO and LUNTO at S_1 or T_1 states can be expressed in equations (S17)~(S20), respectively

$$r_{\mathrm{H}'}^{\mathrm{S}} = \sum_{k}^{all} r_k f_{H'}^{S}(r_k) / \sum_{k}^{all} f_{H'}^{S}(r_k) \tag{S17}$$

$$r_{\mathrm{L}'}^{\mathrm{S}} = \sum_{k}^{all} r_k f_{L'}^{S}(r_k) / \sum_{k}^{all} f_{L'}^{S}(r_k) \tag{S18}$$

$$r_{\mathrm{H}'}^{\mathrm{T}} = \sum_{k}^{all} r_k f_{H'}^{T}(r_k) / \sum_{k}^{all} f_{H'}^{T}(r_k) \tag{S19}$$

$$r_{\mathrm{L}'}^{\mathrm{T}} = \sum_{k}^{all} r_k f_{L'}^{T}(r_k) / \sum_{k}^{all} f_{L'}^{T}(r_k) \tag{S20}$$

Thus, the mean distances between HONTO and LUNTO at S_1 or T_1 states are

$$< r_{\mathrm{S}} > = \left| r_{H'}^{S} - r_{L'}^{S} \right| \tag{S21}$$

$$< r_{\mathrm{T}} > = \left| r_{H'}^{T} - r_{L'}^{T} \right| \tag{S22}$$

The values of I_{S}, I_{T}, $<r_{\mathrm{S}}>$, and $<r_{\mathrm{T}}>$ have considered the whole picture of the electron interactions of the corresponding excited states, providing more information and physical insights of the excited states than $I_{\mathrm{H/L}}$ and $<r_{\mathrm{H/L}}>$.

All in all, in principle, the ΔE_{ST} can be expressed in equation (S5) and can be simplified to equation (S13). From equation (S5) and (S13), the ΔE_{ST} is dependent on the frontier orbital overlap extent and separation distance at S_0 state. The higher overlap of HOMO and LUMO and smaller spatial separation (r_1-r_2) lead to higher ΔE_{ST}.

From the 35 compounds except for **A5** and **A6**, the average ΔE_{ST} / ($I_{\mathrm{H/L}}^2$ / $<r_{\mathrm{H/L}}>$) is 25.7, which is very close to 28.8 in equation (S13). The very high values observed in **A5** and **A6** clearly suggest the unfitness of the conventional HOMO-LUMO transition analysis, especially when there are very low HOMO → LUMO transition components for their T_1 states. Therefore, it is not accurate to address that ΔE_{ST} is only determined by $I_{\mathrm{H/L}}$ and $<r_{\mathrm{H/L}}>$ as in equation (S13), overlooking all the different transitions other than HOMO → LUMO. Therefore, with the aid of NTO analysis, we use a set of new parameters of I_{S}, I_{T}, $<r_{\mathrm{S}}>$, and $<r_{\mathrm{T}}>$ to develop equation (S23)

$$\Delta E_{\mathrm{ST}} = C_{\mathrm{S}} \frac{I_S^2}{\langle r_S \rangle} + C_{\mathrm{T}} \frac{I_T^2}{\langle r_T \rangle} \qquad \text{(S23)}$$

For the all the 37 studied compounds, ΔE_{ST} / (I_{S}^2 / < r_{S} > + I_{T}^2 / < r_{T} >) varies in a relatively narrow range from 0.15 to 11.16, indicating that equation (S23) presents a good correlation between ΔE_{ST} and parameters of I_{S}, I_{T}, < r_{S} >, and < r_{T} >. Thus, in studying ΔE_{ST}, it is more advisable to consider the HONTO and LUNTO at both S_1 and T_1 states.

4. Supplementary figures

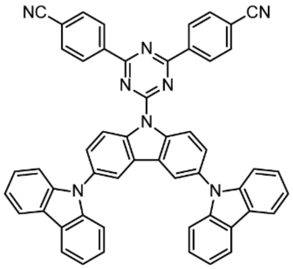

A11

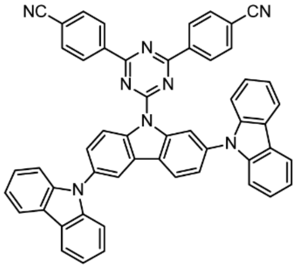

A12

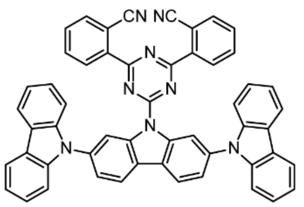

A13

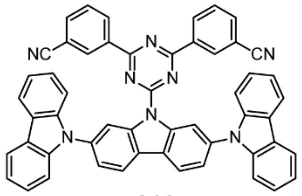

A14

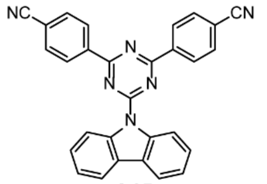

A15

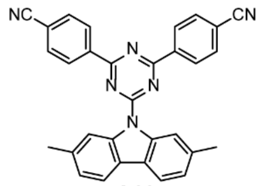

A16

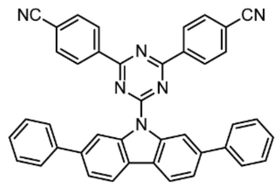

A17

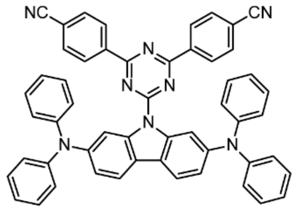

A18

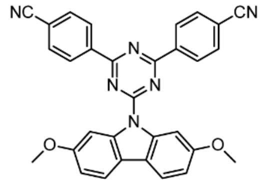

A19

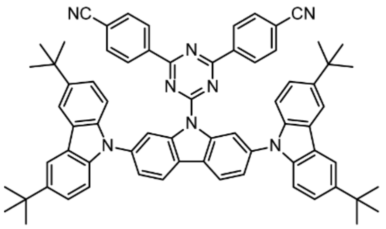

A20

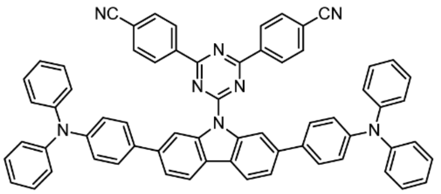

A21

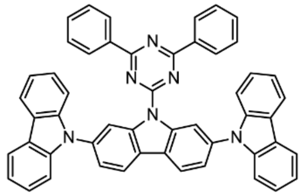

A22

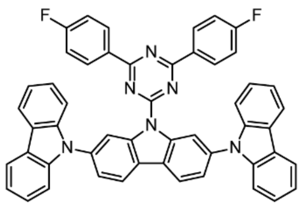

A23

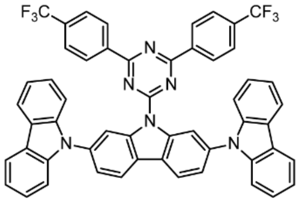

A24

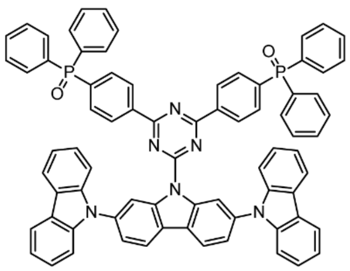

A25

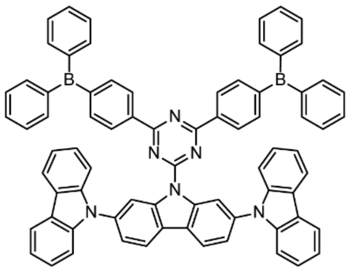

A26

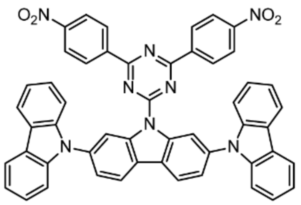

A27

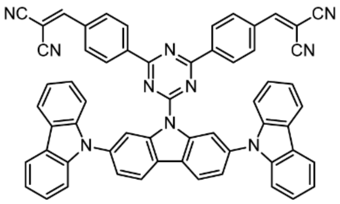

A28

Figure S1. The asymmetric triazines of **A11~A28**.

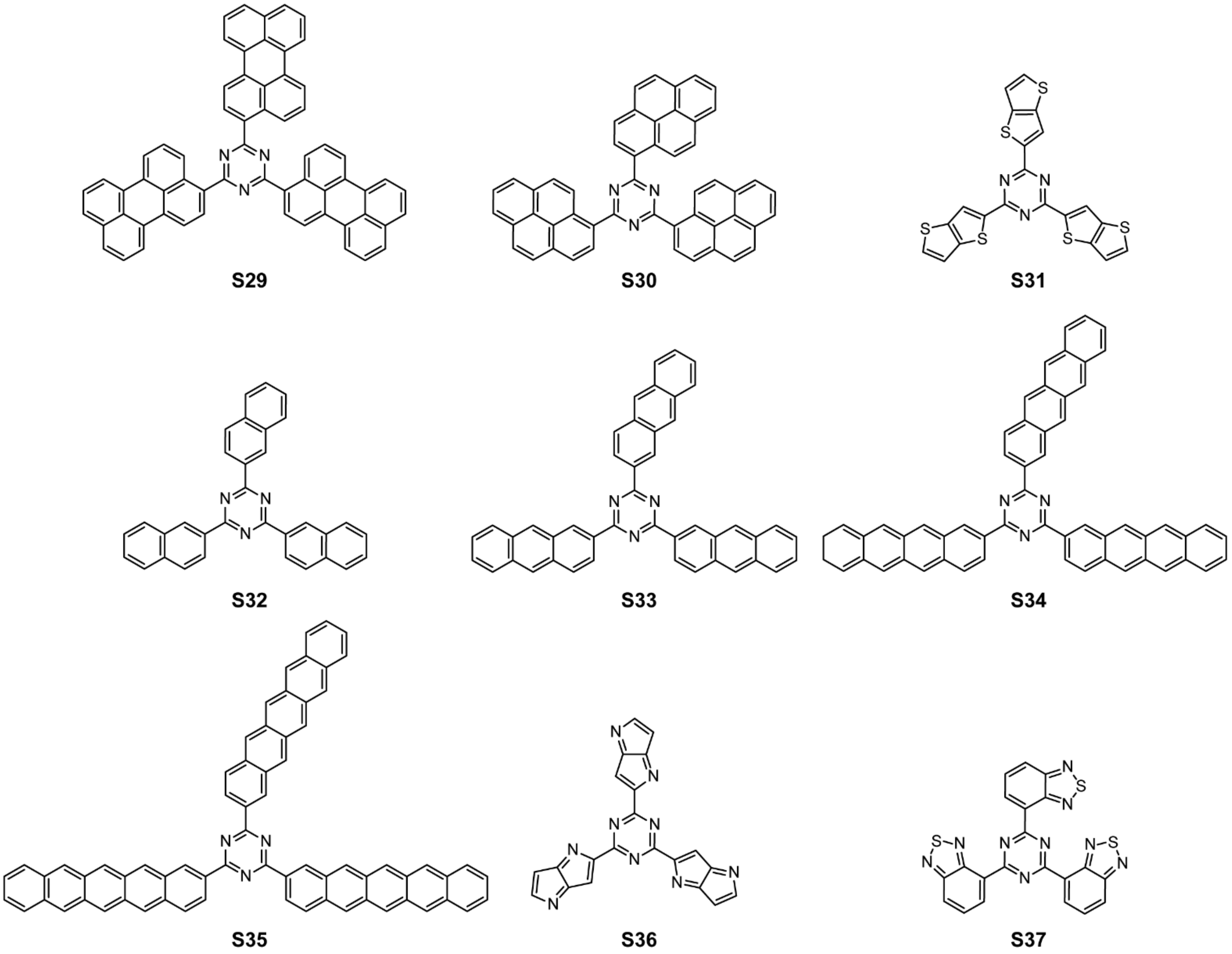

Figure S2. The symmetric triazines of **S29~S37**.

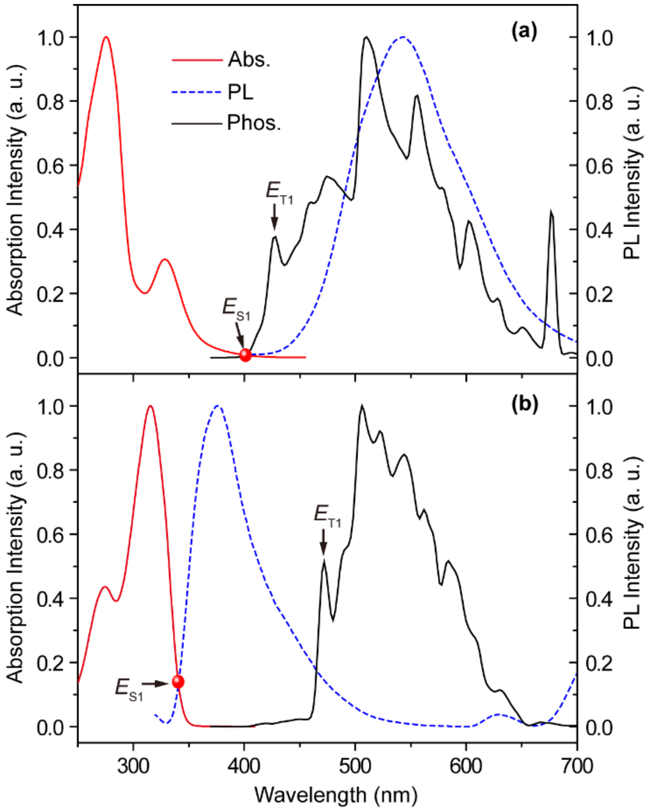

Figure S3. Experimental investigations on photophysical properties of triazine derivatives of **(a) A15** and **(b) S9**. Note that the absorption and emission spectra of **A15** and **S9** were measured in dilute chloroform at room temperature. The phosphorescence spectrum measurements were performed in chloroform at 77 K after 5 ms delay of the excitation at 330 nm to eliminate the strong but short-lived fluorescence.

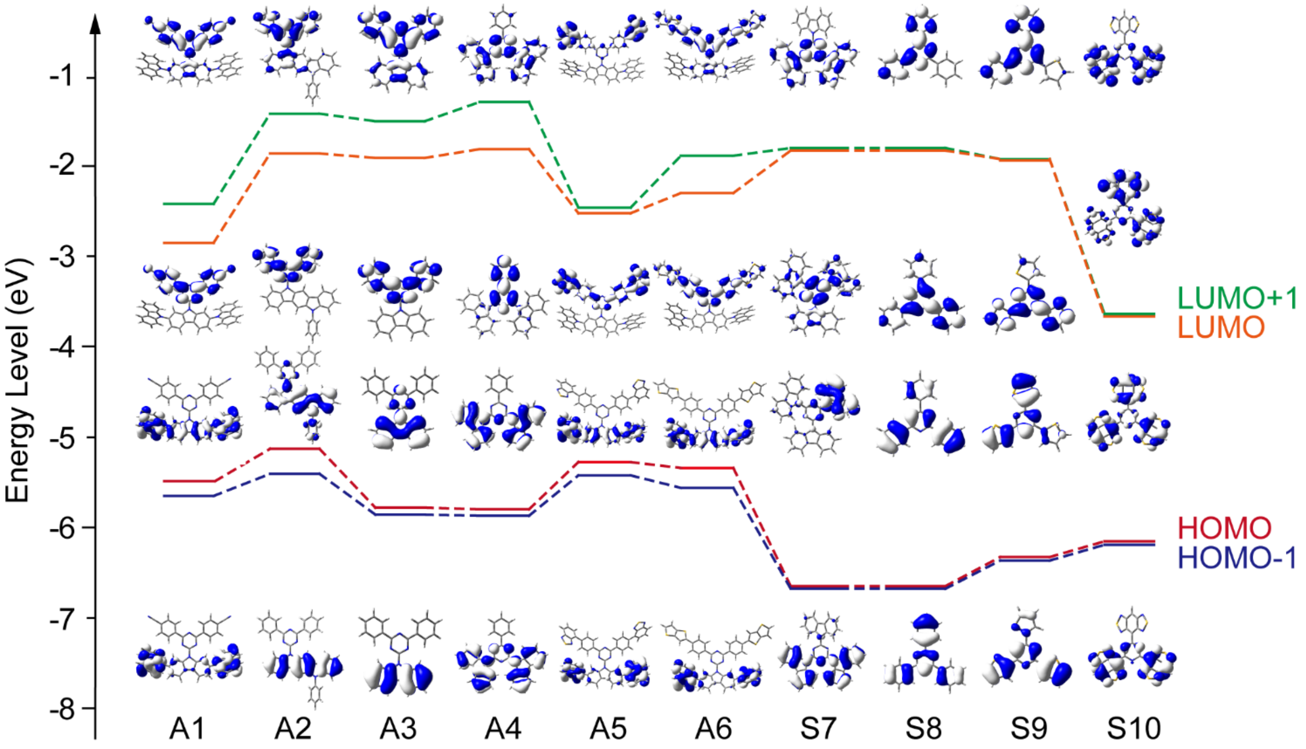

Figure S4. The calculated energy levels of HOMO-1, HOMO, LUMO and LUMO+1 and their spatial plot (isovalue 0.02) of **A1**~**A6** and **S7**~**S10**.

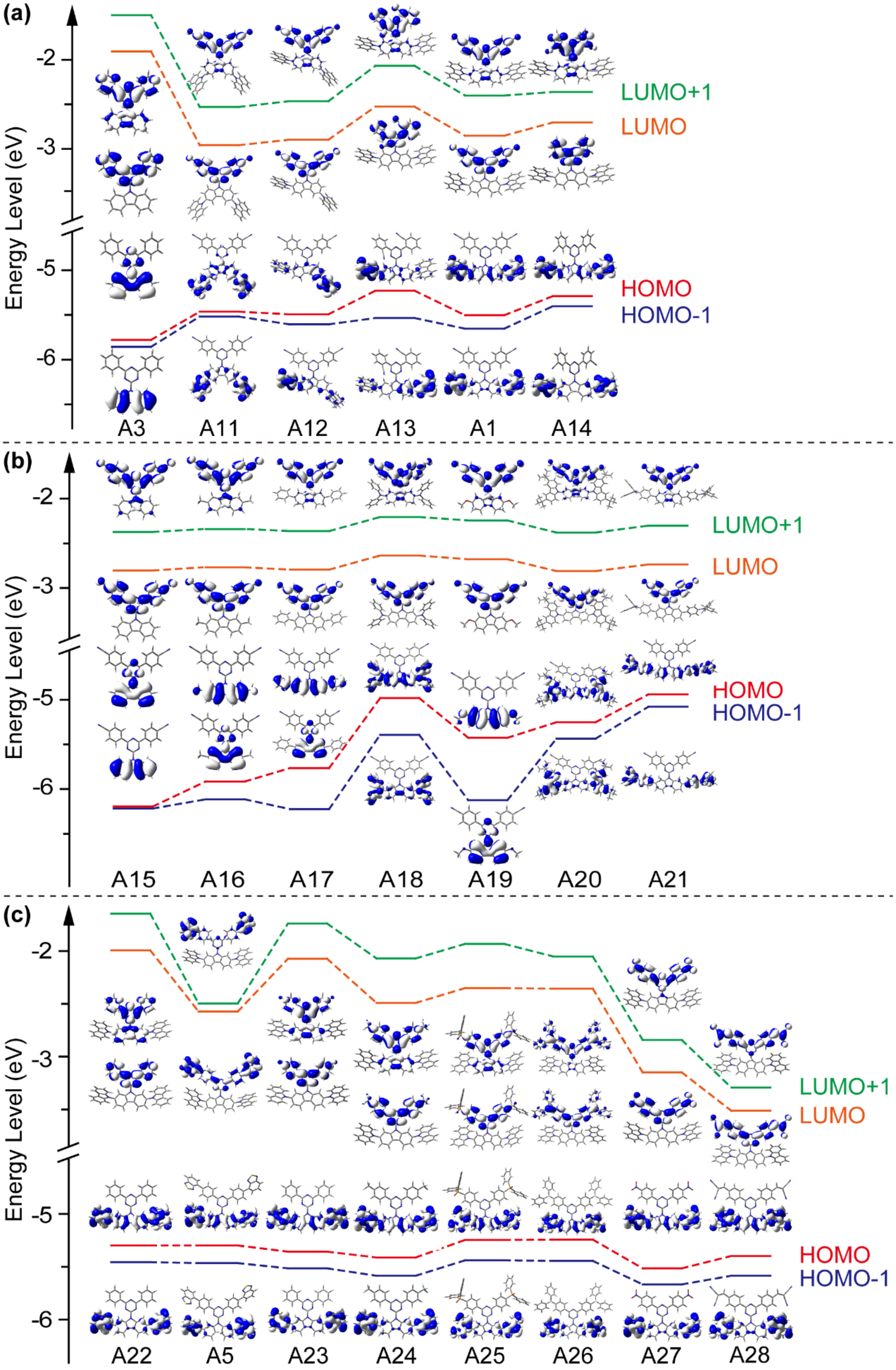

Figure S5. The calculated energy levels of HOMO-1, HOMO, LUMO and LUMO+1 and their spatial plot (isovalue 0.02) of **(a) A3** and **A11~A14**, **(b) A15~A21**, **(c) A5**, and **A22~A28**.

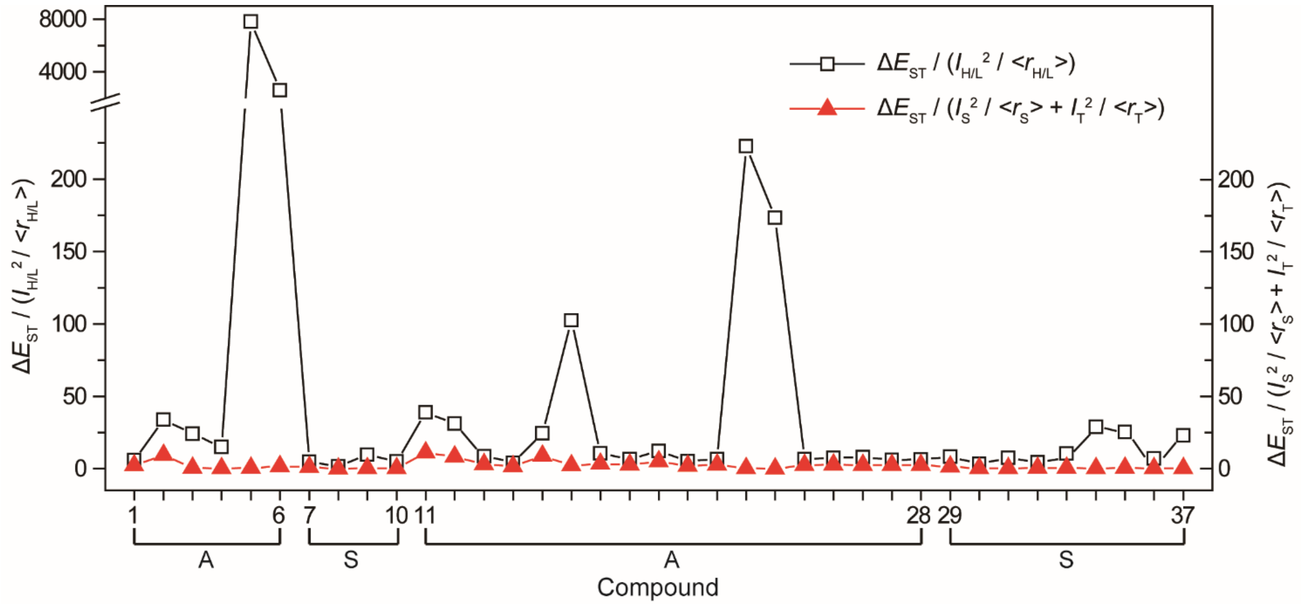

Figure S6. The values of ΔE_{ST} / ($I_{H/L}^2$ / <$r_{H/L}$>) and ΔE_{ST} / (I_S^2 / <r_S>+ I_T^2 / <r_T>) of **A1~S37**.

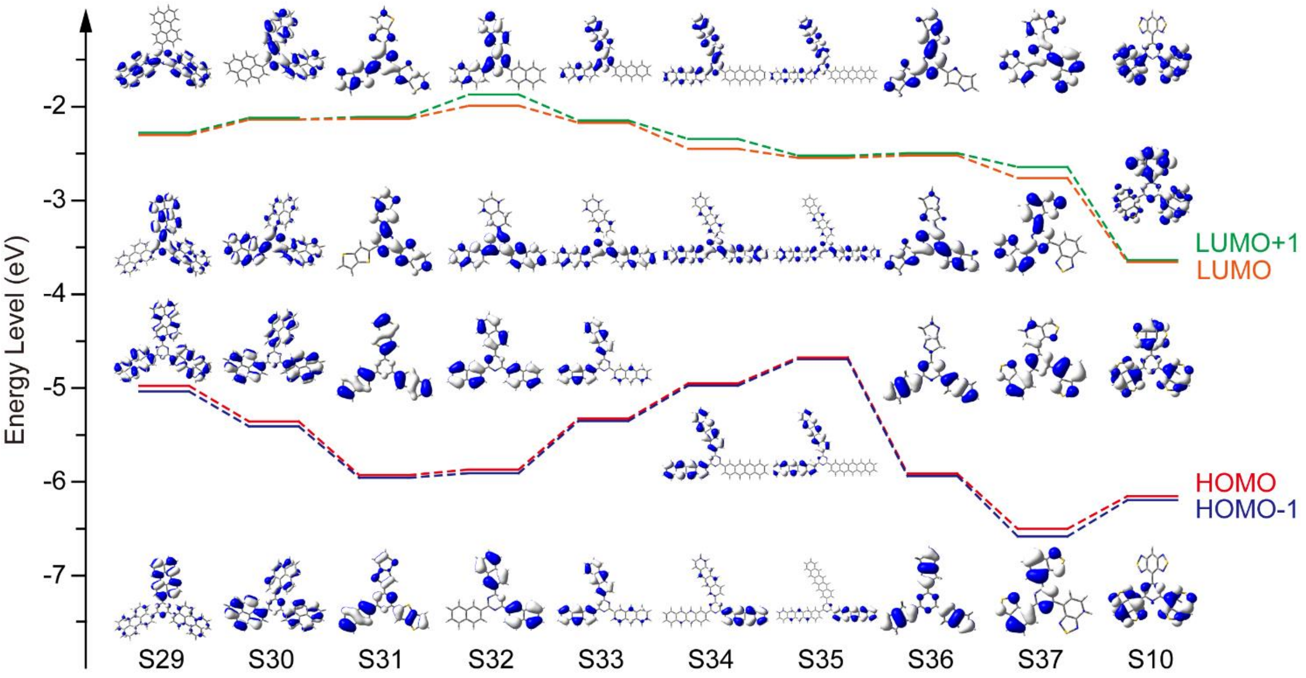

Figure S7. The calculated energy levels of HOMO-1, HOMO, LUMO and LUMO+1 and their spatial plot (isovalue 0.02) of **S10** and **S29~S37**.

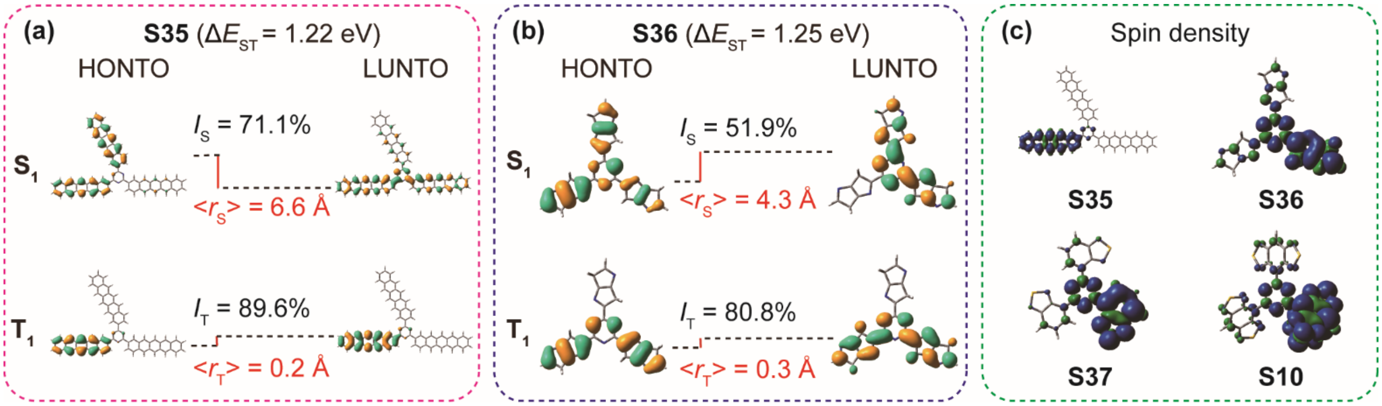

Figure S8. The highest occupied NTO (HONTO) and lowest unoccupied NTO (LUNTO) according to the results of TD-DFT calculations for the S_1 and T_1 states of **S35 (a)**, **S36 (b)** on the optimized structure at their ground states (S_0); **(c)** The spin density (isovalue 0.0004) of the optimized T_1 states for **S35~S37** and **S10**.

5. Supplementary tables

Table S1. The molecular structures, energies (E in eV) of S_1 and T_1, S_1-T_1 splitting (ΔE_{ST} in eV), configuration interaction (CI), and frontier orbital overlap extents ($I_{H/L}$, I_S, and I_T) and mean orbital separation distances ($\langle r_{H/L}\rangle$, $\langle r_S\rangle$, and $\langle r_T\rangle$ in Å) of compounds experimentally studied by Adachi and co-workers

Compound		E (eV)	CI description[a]	ΔE_{ST} [b]	$I_{H/L}$ / $\langle r_{H/L}\rangle$	I_S / $\langle r_S\rangle$	I_T / $\langle r_T\rangle$
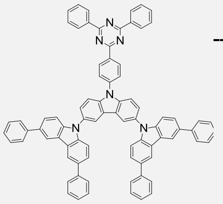 **1**	S_1	2.6704	H → L (98.65%)	0.0618<br>0.05~0.12	12.78% / 18.38	16.80% / 17.06	33.63% / 14.66
	T_1	2.6086	H → L (86.35%)				
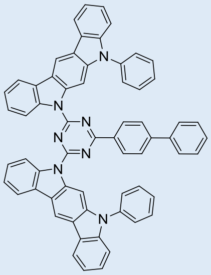 **2**	S_1	2.8439	H → L (97.73%)	0.0781<br>0.11	21.18% / 11.2	25.57% / 10.0	36.40% / 8.0
	T_1	2.7658	H-1→ L (71.45%)				
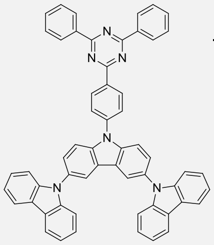 **3**	S_1	2.7651	H → L (98.31%)	0.1167<br>0.19	16.98% / 16.17	21.59% / 14.85	44.40% / 11.53
	T_1	2.6484	H → L (77.89%)				
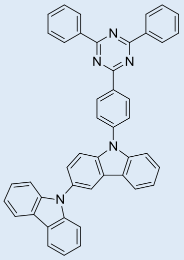 **4**	S_1	2.8105	H → L (97.69%)	0.1371<br>0.29	15.33% / 17.06	20.83% / 15.34	49.29% / 10.42
	T_1	2.6734	H-2→ L (21.18%)<br>H → L (64.87%)				

[a]: the excitation transitions with relative weights > 15%

[b]: the red number is the experimental result[12]

Table S2. The molecular structures, S_1-T_1 splitting (ΔE_{ST} in eV), and frontier orbital overlap extents ($I_{H/L}$, I_S, and I_T) and mean separation distances ($\langle r_{H/L}\rangle$, $\langle r_S\rangle$, and $\langle r_T\rangle$ in Å) studied by B3LYP, PBE0, BMK and M062X methods at the basis set level of 6-31G(d).

Compound	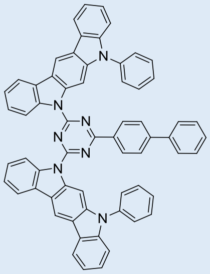 **2**			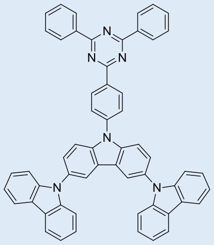 **3**		
	ΔE_{ST}	$I_{H/L}/I_S/I_T$	$\langle r_{H/L}\rangle/\langle r_S\rangle/\langle r_T\rangle$	ΔE_{ST}	$I_{H/L}/I_S/I_T$	$\langle r_{H/L}\rangle/\langle r_S\rangle/\langle r_T\rangle$
B3LYP	0.08	21%/26%/36%	11.2/10.0/8.0	0.12	17%/22%/44%	16.2/14.8/11.5
PBE0	0.25	21%/28%/80%	11.2/9.6/0.8	0.27	17%/24%/65%	16.1/14.2/7.8
BMK	0.49	21%/37%/80%	11.2/7.6/0.2	0.49	17%/37%/74%	16.1/11.5/5.2
M062X	0.53	21%/44%/78%	11.2/5.7/0.2	0.56	18%/47%/75%	16.0/9.2/4.5
Exp.[12]	0.11	-		0.19	-	

Table S3. The energies (E) of S_1 and T_1, S_1-T_1 splitting (ΔE_{ST}), configuration interaction (CI), and frontier orbital overlap extents ($I_{H/L}$, I_S, and I_T) and mean separation distances ($<r_{H/L}>$, $<r_S>$, and $<r_T>$) of **A1~S10**

Compound	E (eV)		CI description[a]	ΔE_{ST} (eV)	$I_{H/L}$	$<r_{H/L}>$ (Å)	I_S	$<r_S>$ (Å)	I_T	$<r_T>$ (Å)
**A1**	S_1	2.2963	H → L (99.29%)	0.0011	4.23%	9.52	4.51%	9.45	5.15%	9.37
	T_1	2.2952	H → L (99.28%)							
**A2**	S_1	2.8209	H → L (98.24%)	0.0893	17.43%	11.48	19.07%	10.86	24.70%	10.03
	T_1	2.7316	H → L (93.54%)							
**A3**	S_1	3.2854	H → L (99.08%)	0.1742	24.31%	8.17	24.82%	7.99	81.48%	2.37
	T_1	3.1112	H-1→ L+2 (37.08%)<br>H → L (35.21%)							
**A4**	S_1	3.4693	H-1 → L (91.35%)	0.3563	35.27%	5.21	41.84%	4.43	81.95%	0.79
	T_1	3.1130	H-3 → L+2 (25.26%)<br>H → L (35.21%)							
**A5**	S_1	2.4051	H → L (85.74%)	0.386	2.57%	13.35	2.96%	12.64	84.25%	1.08
	T_1	2.0191	H-4 → L+1 (32.12%)<br>H-4 → L (29.03%)<br>H-6 → L (15.39%)							
**A6**	S_1	2.6783	H → L (99.01%)	0.4606	4.44%	11.18	4.76%	10.98	79.97%	2.25
	T_1	2.2177	H-3 → L (35.80%)<br>H-2 → L (17.76%)<br>H-2 → L+2 (24.09%)							
**S7**	S_1	3.7333	H → L (94.13%)	0.6347	50.97%	1.88	51.40%	7.34	81.14%	1.31
	T_1	3.0986	H-4 → L+1 (18.72%)							
**S8**	S_1	4.0718	H-7 → L+1 (48.62%)<br>H-7 → L (49.74%)	1.0492	73.85%	0.85	41.84%	0.24	81.95%	0.10
	T_1	3.0226	H → L (35.13%)<br>H-1 → L+1 (35.05%)							
**S9**	S_1	3.6920	H-1 → L+1 (44.28%)<br>H → L (54.33%)	1.0809	56.63%	2.90	55.65%	2.98	79.92%	0.24
	T_1	2.6111	H-1 → L (36.66%)<br>H → L+1 (46.86%)							
**S10**	S_1	2.1413	H-2 → L+1 (25.11%)<br>H-1 → L (66.64%)	1.4690	76.00%	2.00	50.70%	5.07	83.17%	0.18
	T_1	0.6723	H-2 → L+1 (19.07%)<br>H-1 → L (19.35%)<br>H-1 → L+2 (36.10%)<br>H → L+1 (44.10%)<br>H-1 ← L (3.53%)<br>H-1 ← L+2 (6.95%)<br>H ← L+1 (7.86%)							

[a]: the excitation transitions with relative weights > 15% and the de-excitation transitions;

Table S4. The energies (E) of S_1 and T_1, S_1-T_1 splitting (ΔE_{ST}), configuration interaction (CI), and frontier orbital overlap extents ($I_{H/L}$, I_S, and I_T) and mean separation distances ($<r_{H/L}>$, $<r_S>$, and $<r_T>$) of **A11~A28**

Compound		E (eV)	CI description[a]	ΔE_{ST} (eV)	$I_{H/L}$	$<r_{H/L}>$ (Å)	I_S	$<r_S>$ (Å)	I_T	$<r_T>$ (Å)
**A11**	S_1	2.1811	H → L (99.12%)	0.0255	9.94%	15.05	11.35%	14.64	14.14%	14.23
	T_1	2.1556	H → L (97.72%)							
**A12**	S_1	2.2625	H → L (89.23%)	0.0123	7.53%	14.28	8.34%	12.49	10.71%	12.47
	T_1	2.2502	H → L (92.44%)							
**A13**	S_1	2.2582	H → L (98.93%)	0.0011	3.73%	11.30	4.08%	10.73	4.58%	10.66
	T_1	2.2571	H → L (98.97%)							
**A14**	S_1	2.2002	H → L (99.52%)	0.0004	2.91%	9.08	3.14%	9.03	3.60%	8.99
	T_1	2.1998	H → L (99.52%)							
**A15**	S_1	2.8510	H → L (99.28%)	0.1416	22.84%	8.98	23.09%	8.84	28.50%	8.22
	T_1	2.7094	H → L (97.23%)							
**A16**	S_1	2.6893	H → L (99.79%)	0.0234	5.00%	10.94	5.14%	10.83	28.32%	8.24
	T_1	2.6659	H-1 → L (97.23%)							
**A17**	S_1	2.5596	H → L (99.75%)	0.0022	4.64%	10.36	4.73%	10.29	6.54%	10.06
	T_1	2.5574	H → L (99.64%)							
**A18**	S_1	1.9363	H → L (99.59%)	0.0020	5.50%	10.01	5.71%	9.92	6.44%	9.82
	T_1	1.9343	H → L (99.59%)							
**A19**	S_1	2.3040	H → L (99.83%)	0.0019	4.18%	11.34	4.23%	11.25	5.01%	11.16
	T_1	2.3021	H → L (99.82%)							
**A20**	S_1	2.0573	H → L (99.34%)	0.0007	3.52%	9.32	3.78%	9.24	4.12%	9.18
	T_1	2.0566	H → L (99.34%)							
**A21**	S_1	1.8633	H → L (99.94%)	0.0007	3.08%	8.86	3.29%	8.92	3.53%	8.89
	T_1	1.8626	H → L (99.95%)							
**A22**	S_1	2.8989	H → L (99.51%)	0.0548	4.65%	8.79	5.11%	8.73	72.59%	1.62
	T_1	2.8441	H → L+2 (34.23%)<br>H → L+1 (32.97%)							
**A23**	S_1	2.8773	H → L (99.52%)	0.0438	4.68%	8.67	5.12%	8.61	71.68%	1.71
	T_1	2.8335	H → L+1 (34.17%)<br>H → L+2 (34.03%)							
**A24**	S_1	2.5366	H → L (99.46%)	0.0014	4.42%	9.07	4.72%	9.01	5.57%	8.91
	T_1	2.5352	H → L (99.44%)							
**A25**	S_1	2.4988	H → L (99.46%)	0.0010	3.63%	9.99	3.97%	9.87	4.55%	9.80
	T_1	2.4978	H → L (99.45%)							
**A26**	S_1	2.5136	H → L (98.81%)	0.0008	3.40%	11.45	3.78%	11.22	4.58%	11.12
	T_1	2.5128	H → L (98.80%)							
**A27**	S_1	1.9920	H → L (98.25%)	0.0008	3.66%	10.20	3.91%	10.08	4.29%	10.03
	T_1	1.9912	H → L (98.24%)							
**A28**	S_1	1.5352	H → L (97.81%)	0.0003	2.27%	11.03	2.44%	10.93	2.69%	10.91
	T_1	1.5349	H → L (97.82%)							

[a]: the excitation transitions with relative weights > 15%

Table S5. The energies (E) of S_1 and T_1, S_1-T_1 splitting (ΔE_{ST}), configuration interaction (CI), and frontier orbital overlap extents ($I_{H/L}$, I_S, and I_T), and mean separation distances ($<r_{H/L}>$, $<r_S>$, and $<r_T>$) of **S29~S37**

Compound	E (eV)		CI description[a]	ΔE_{ST} (eV)[b]	$I_{H/L}$	$<r_{H/L}>$ (Å)	I_S	$<r_S>$ (Å)	I_T	$<r_T>$ (Å)
**S29**	S_1	2.3514	H → L (97.30%)	0.918	67.59%	4.19	61.45%	4.70	88.37%	1.22
	T_1	1.4334	H-1 → L (55.77%)	0.61						
			H → L+2 (17.18%)							
**S30**	S_1	2.8372	H-2 → L (40.84%)	0.9181	72.60%	1.84	23.54%	10.62	86.19%	0.24
			H-1 → L+1 (28.74%)	0.68						
	T_1	1.9191	H-2 → L+1 (32.45%)							
			H-1 → L (36.48%)							
			H → L+2 (22.07%)							
**S31**	S_1	3.2627	H-2 → L+1 (48.62%)	0.9625	60.54%	2.84	51.86%	4.43	80.78%	0.18
			H-1 → L (49.09%)	0.70						
	T_1	2.3002	H-2 → L (40.33%)							
			H-1 → L+1 (40.47%)							
**S32**	S_1	3.4411	H-1 → L (48.26%)	0.9963	68.15%	2.09	35.48%	6.96	84.79%	0.33
			H → L (48.26%)	0.71						
	T_1	2.4448	H → L (39.46%)							
**S33**	S_1	2.7365	H-1 → L (22.43%)	1.05	66.98%	4.74	49.77%	7.17	86.87%	0.34
			H → L (72.50%)	0.62						
	T_1	1.6865	H-1 → L+2 (16.01%)							
			H → L (30.19%)							
**S34**	S_1	2.1992	H → L (89.91%)	1.1247	59.56%	9.11	59.80%	7.44	89.00%	0.28
	T_1	1.0745	H-2 → L+1 (21.00%)	0.49						
			H-1 → L (20.44%)							
			H → L (20.91%)							
**S35**	S_1	1.7827	H → L (96.91%)	1.2219	66.60%	8.98	71.07%	6.64	89.64%	0.27
	T_1	0.5608	H-2 → L+1 (28.37%)	0.31						
			H-1 → L (26.15%)							
			H → L (16.97%)							
			H → L+2 (24.93%)							
**S36**	S_1	2.8744	H-2 → L (34.35%)	1.2486	65.44%	1.76	50.81%	4.30	83.55%	0.30
			H-1 → L+1 (34.37%)	0.57						
	T_1	1.6258	H-2 → L+1 (27.49%)							
			H-1 → L (27.59%)							
			H → L+2 (20.72%)							
**S37**	S_1	3.3293	H-1 → L+1 (48.20%)	1.2787	52.65%	5.00	54.86%	4.50	85.04%	0.23
			H → L (48.92%)	0.62						
	T_1	2.0506	H-2 → L+2 (25.19%)							
			H-1 → L (35.37%)							
			H → L+1 (35.37%)							

[a]: the excitation transitions with relative weights > 15%; [b]: the red number is the value of E_{T1}/E_{S1}

References:

1. An, Z. et al. Conjugated asymmetric donor-substituted 1,3,5-triazines: new host materials for blue phosphorescent organic light-emitting diodes. *Chem. Eur. J.* **17**, 10871-10878 (2011).
2. An, Z. et al. Exceptional blueshifted and enhanced aggregation-induced emission of conjugated asymmetric triazines and their applications in superamplified detection of explosives. *Chem. Eur. J.* **18**, 15655-15661 (2012).
3. Huang, S. et al. Computational prediction for singlet- and triplet-transition energies of charge-transfer compounds. *J. Chem. Theory Comput.* **9**, 3872-3877 (2013).
4. Tao, Y. et al. Dynamically adaptive characteristics of resonance variation for selectively enhancing electrical performance of organic semiconductors. *Angew. Chem. Int. Edit.* **52**, 10491-10495 (2013).
5. Fan, M. G. & Yao, J. N. *Photochemistry and optical function materials* Science Press, 2009
6. Endo, A. et al. Thermally activated delayed fluorescence from sn^{4+}-porphyrin complexes and their application to organic light emitting diodes-a novel mechanism for electroluminescence. *Adv. Mater.* **21**, 4802-4806 (2009).
7. Busmann, H. G., Staerk, H. & Weller, A. Solvent influence on the magnetic field effect of polymethylene-linked photogenerated radical ion pairs. *J. Chem. Phys.* **91**, 4098-4105 (1989).
8. Lu, T. & Chen, F. Multiwfn: a multifunctional wavefunction analyzer. *J. Comput. Chem.* **33**, 580-592 (2012).
9. Lu, T. *Multiwfn: a multifunctional wavefunction analyzer* version 3.3.3, 2013
10. Kim, D., Coropceanu, V. & Brédas, J. Design of efficient ambipolar host materials for organic blue electrophosphorescence: theoretical characterization of hosts based on carbazole derivatives. *J. Am. Chem. Soc.* **133**, 17895-17900 (2011).
11. Martin, R. L. Natural transition orbitals. *J. Chem. Phys.* **118**, 4775-4777 (2003).
12. Hirata, S. et al. Highly efficient blue electroluminescence based on thermally activated delayed fluorescence. *Nat. Mater.* **14**, 330-336 (2015).
